# Expression of 5‐methylcytosine regulators is highly associated with the clinical phenotypes of prostate cancer and *DNMTs* expression predicts biochemical recurrence

**DOI:** 10.1002/cam4.4108

**Published:** 2021-07-05

**Authors:** Lin Wang, Guoping Ren, Biaoyang Lin

**Affiliations:** ^1^ College of Life Science Zhejiang University Hangzhou China; ^2^ Systems Biology Division, Zhejiang California International Nanosystems Institute (ZCNI) Zhejiang University Hangzhou China; ^3^ Department of Pathology, The First Affiliated Hospital, School of Medicine Zhejiang University Hangzhou China; ^4^ Collaborative Innovation Center for Diagnosis and Treatment of Infectious Diseases, The First Affiliated Hospital, School of Medicine Zhejiang University Hangzhou China; ^5^ Department of Urology University of Washington Seattle Washington USA

**Keywords:** biochemical recurrence, DNA methyltransferase, immune infiltration, prostate cancer

## Abstract

In patients with prostate cancer (PCa), there is a high rate of overdiagnosis and frequent overtreatment. Therefore, there is an urgent need for more accurate prediction of biochemical recurrence (BCR). DNA methylation regulation patterns play crucial roles in tumorigenicity, progression, and treatment efficacy in PCa. However, the global relationship between epigenetic alterations, changes in mRNA levels, and pathologic phenotypes of PCa remain largely undefined. Here, we conducted a systematic analysis to identify global coexpression and comethylation modules in PCa. We identified coregulated methylation and expression modules and the relationships between epigenetic modifications, tumor progression, and the corresponding immune microenvironment in PCa. Our results show that DNA methyltransferases (DNMTs) are strongly associated with pathologic phenotypes and immune infiltration patterns in PCa. We built a two‐factor predictive model using the expression features of *DNMT3B* and *DNMT1*. The model was used to predict the BCR status of patients with PCa and achieved area under the receiver operating characteristic curve values of 0.70 and 0.88 in the training and independent testing datasets, respectively.

## Graphical abstract

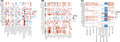

## 1 INTRODUCTION

Prostate cancer (PCa) is one of the most common cancers in males, and in 2020 is estimated to account for more than one in five new cancer diagnoses in men in the United States.^1^ Although most PCas remain indolent, some show aggressive and metastatic phenotypes. For example, castration‐resistant prostate cancer (CRPC) is the main cause of PCa death and has few therapeutic treatment options. The heterogeneous nature of PCa means that it is important that an accurate outcome prediction model is developed so that appropriate treatments can be applied and overtreatment can be avoided. The Gleason score remains one of the most powerful prognostic predictors for PCa. The Gleason grading system defines five histological grades, with Gleason 1 representing the most differentiated PCa and Gleason 5 representing the least differentiated PCa. Many PCas harbor more than one Gleason pattern, so the final Gleason score represents the sum of the primary and secondary patterns and better correlates with biological behavior and prognosis.^2^

The epigenetic machinery is composed of four main components: DNA methylation, N6‐methyladenosine (m6A) RNA modification, histone modification, and noncoding RNAs (ncRNAs). DNA methylation is one of the most common epigenetic events and is involved in various physiological and pathological processes, including genomic imprinting, X chromosome inactivation, tissue‐specific gene expression, chromosome stability, transposable element repression, aging, and diseases including cancer.^3^, ^4^ DNA methylation is a crucial epigenetic modification in mammalian cells and is catalyzed by the addition of a methyl group to the C‐5 position of cytosine residues at CpG nucleotides by DNA methyltransferases (DNMTs) to form 5mC.^5^ Methylated DNA is interpreted by readers, including methyl‐CpG‐binding domain (MBD) family proteins, Kaiso family proteins, and SET‐ and Ring finger‐associated (SRA) domain family proteins.^6^ DNA methylation can be further edited by ten‐eleven translocation (TET) protein family proteins, which oxidize 5mC into 5‐hydroxymethylcytosine (5‐hmC).^7^ DNA hypomethylation and hypermethylation events have been extensively described in carcinogenesis and tumor progression, including in PCa.^8^, ^9^, ^10^ Aberrant DNA methylation of *GSTP1* and *HOX* family genes recurrently occurs in PCa.^11^, ^12^ Additionally, DNA methylation markers can be easily detected in liquid biopsy samples, including in urine and blood, making them excellent noninvasive biomarkers for both diagnosing and monitoring disease progression.^13^ Besides, DNMTS were shown to affect the immune response of variety of human cancer. Hypomethylating agents in breast cancer was shown to involve in upregulating class‐I antigen presentation to potentiate CD8^+^ T cell responses.^14^ Hypermethylation limited immune checkpoint blockade (CPB) immunotherapy by inhibiting interferon responses while global hypomethylation caused upregulation of PD‐L1 and inhibitory cytokines, accompanied by epithelial‐mesenchymal changes that can contribute to immunosuppression.^15^

Here, we focused on genes negatively regulated by promoter methylation status. We also sought to identify epigenetically regulated coexpression modules in patients with PCa using data from The Cancer Genome Atlas (TCGA) and weighted gene coexpression network analysis (WGCNA).^16^ By combining methylation and expression profiles, we gained a better understanding of how DNA methylation participates in PCa carcinogenesis and modifies pathological phenotypes. We found that DNMT was strongly associated with pathologic phenotypes and immune infiltration patterns in PCa. We further built a predictive model using only DNMT3B and DNMT1 expression features. This model was used to predict the biochemical recurrence (BCR) status of patients with PCa and achieved area under the receiver operating characteristic (ROC) curve values of 0.70 and 0.88 in the training and independent testing datasets, respectively.

## 2 MATERIALS AND METHODS

### 2.1 Data processing and identification of methylation‐regulated genes

Expression in FPKM (Fragments Per Kilobase of transcript per Million mapped reads), Illumina Human Methylation 450 data and mutation profile from the Genomic Data Commons (GDC) TCGA prostate adenocarcinoma (PRAD) datasets were downloaded using the UCSC Xena Browser (https://xenabrowser.net/datapages/). Promoters were defined as regions −2000 to +100 bp of transcription starting sites. Genes with an average FPKM value ≥1 and CpG sites with an average *β* value ≥0.1 were retained. To identify genes negatively regulated by promoter methylation levels, Pearson correlation analysis was used to compare gene expression and promoter methylation levels across PCa samples. Negative regulation pairs were defined as having a *p*‐value <0.05 and a correlation coefficient <0. Mean *β* values were used when there were multiple negatively regulated pairs per gene. A total of 4660 genes and corresponding promoter methylation information were used in the following analysis.

### 2.2 Construction of weighted gene coexpression network

The networks for expression and methylation levels of selected genes were constructed separately using the WGCNA package in R.^16^ An unsigned network, using the absolute Pearson correlation value for both mRNA expression and DNA methylation, was built and transformed into a weighted adjacency matrix. The power value was chosen based on scale‐free topology criterion. A power value of 8 was chosen to produce networks with a scale‐free topology model fit >0.8. Automatic network construction using blockwiseModules with deepSplit = 4 identified 19 mRNA expression modules and 13 DNA methylation modules. ModulePreservation function implemented in WGCNA Bioconductor R package was applied to test preservation levels of the coexpressed and comethylation modules based on the preservation statistics *Z*_summary_. *Z*_summary_ combines multiple statistics into a single overall measure of preservation that considers density and connectivity aspects of preservation using the following formula:

$$Z_{\mathrm{summary}}=\frac{Z_{\mathrm{density}}+Z_{\mathrm{connectivity}}}{2}$$

where $Z_{\mathrm{density}}$ evaluated the connectedness of each gene within modules while $Z_{\mathrm{connectivity}}$ compared the connectivity patterns between genes of the same network. The higher the value of a *Z*_summary_, the stronger the evidence that the module is preserved. *Z*_summary_ values >10 marked the most preservative modules.^17^, ^18^, ^19^

### 2.3 Assessment of tumor‐infiltrating immune cells

Cell‐type identification by estimating relative subsets of RNA transcripts (CIBERSORT) implemented support vector regression (SVR) method to improve deconvolution performance through a combination of feature selection and robust mathematical optimization techniques. Meanwhile, this algorithm was also proved to well characterizing cellular components in microarray or RNA‐seq data derived from fresh, frozen, and fixed specimens.^21^ Thus, CIBERSORT was used to profile the infiltration levels of 22 different types of immune cells in each PCa sample.^20^ In detail, the LM22 leukocyte gene signature matrix, consisting of 22 immune cell types (naïve B cells, memory B cells, plasma cells, CD8^+^ T cells, naïve CD4^+^ T cells, resting memory CD4^+^ T cells, activated memory CD4^+^ T cells, follicular helper T cells, regulatory T cells [Tregs], gamma delta T cells, resting natural killer [NK] cells, activated NK cells, monocytes, M0 macrophages, M1 macrophages, M2 macrophages, resting dendritic cells, activated dendritic cells, resting mast cells, activated mast cells, eosinophils, and neutrophils), was used to perform the analysis.

### 2.4 Constitution of the 5‐methylcytosine regulator‐based risk model for BCR

To build a prognostic signature for BCR in PCa using 5mC regulator expression, we randomly selected 70% of the TCGA data as the training set to build the model. BCR time and status of samples were directly extracted from the TCGA phenotype file. All the 22 genes that regulate DNA 5mC modifications were subjected to the univariate cox regression analysis to extract independent prognostic genes for BCR using survival package in R according to the cutoff criterion of *p*‐value <0.05. The independent prognostic 5‐methylcytosine (5mC) regulators were then selected as final model candidates. The least absolute shrinkage and selection operator (LASSO) Cox regression algorithm was used to calculate model coefficients using 10‐fold cross‐validation. The risk model was constructed as:

$$\mathrm{risk}\;\mathrm{score}=\;\sum_{k=1}^{n}\left({\mathrm{expression}}_{k}\times {\mathrm{coefficient}}_{k}\right)$$

where ${\mathrm{expression}}_{k}$ is the expression value of the genes and ${\mathrm{coefficient}}_{k}$ is the corresponding LASSO Cox regression coefficient. The remaining data were set as the validation set.

### 2.5 Survival and ROC analysis

Kaplan–Meier analysis and univariate and multivariate Cox analyses were performed using the survival and survminer packages in R. ROC and time‐dependent ROC were analyzed using pROC and timeROC, respectively.

### 2.6 Differential expression analysis and functional enrichment analysis

The R package limma^22^ was used to identify genes that were significantly differentially expressed in samples from high‐ and low‐risk groups. Differentially expressed genes were selected with the threshold of |log fold change (FC)| > 1 and an adjusted *p*‐value <0.01. From the gene set perspective, gene set enrichment analysis (GSEA) was used to detect significant differences between high‐ and low‐risk groups using MSigDB hallmark gene sets (h.all.v7.0.symbols.gmt). GSEA was performed using the R package fgsea with 100 permutations for each analysis.^23^ Pathways with the top five smallest *p*‐values for both positively and negatively enriched sets are shown in the results. The R package clusterProfiler was used to perform Gene Ontology (GO) and Kyoto Encyclopedia of Genes and Genomes (KEGG) pathway enrichment analyses for differentially expressed genes.^24^ A false discovery rate (FDR) adjusted *p*‐value <0.05 was considered statistically significant for GO and KEGG pathway enrichment analyses.

### 2.7 Construction of miRNA‐5mC regulator regulation network

The potential miRNA‐5mC regulator regulation relationships were retrieved from the online database miRNet.^25^ After then Pearson correlation analysis was applied between expression levels of each potential pairs and those with correlation coefficient <−0.3 and *p*‐value <0.01 were defined as the final candidates.

## 3 RESULTS

### 3.1 Identification of coexpression and comethylation modules in PCa

**FIGURE 1:**
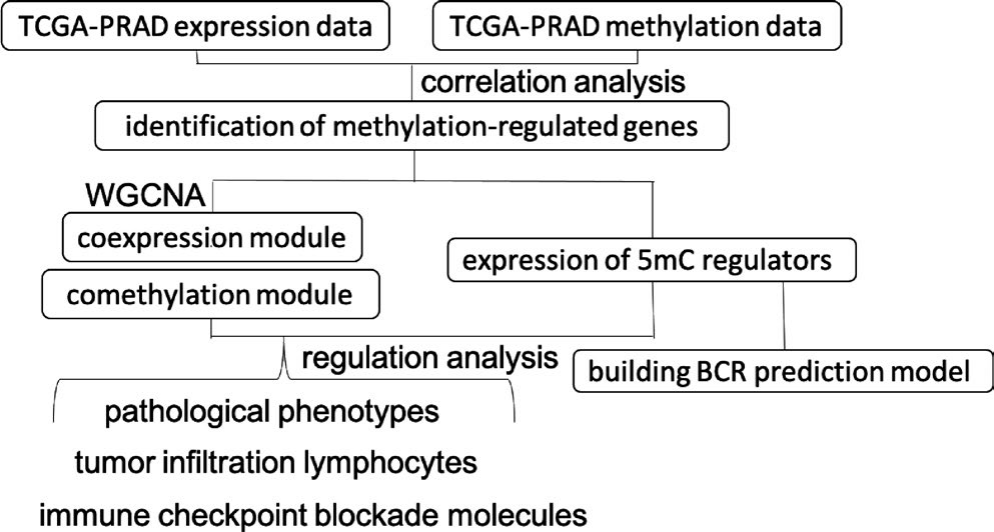
A schematic diagram depicting the analysis pipeline of this study

**FIGURE 2:**
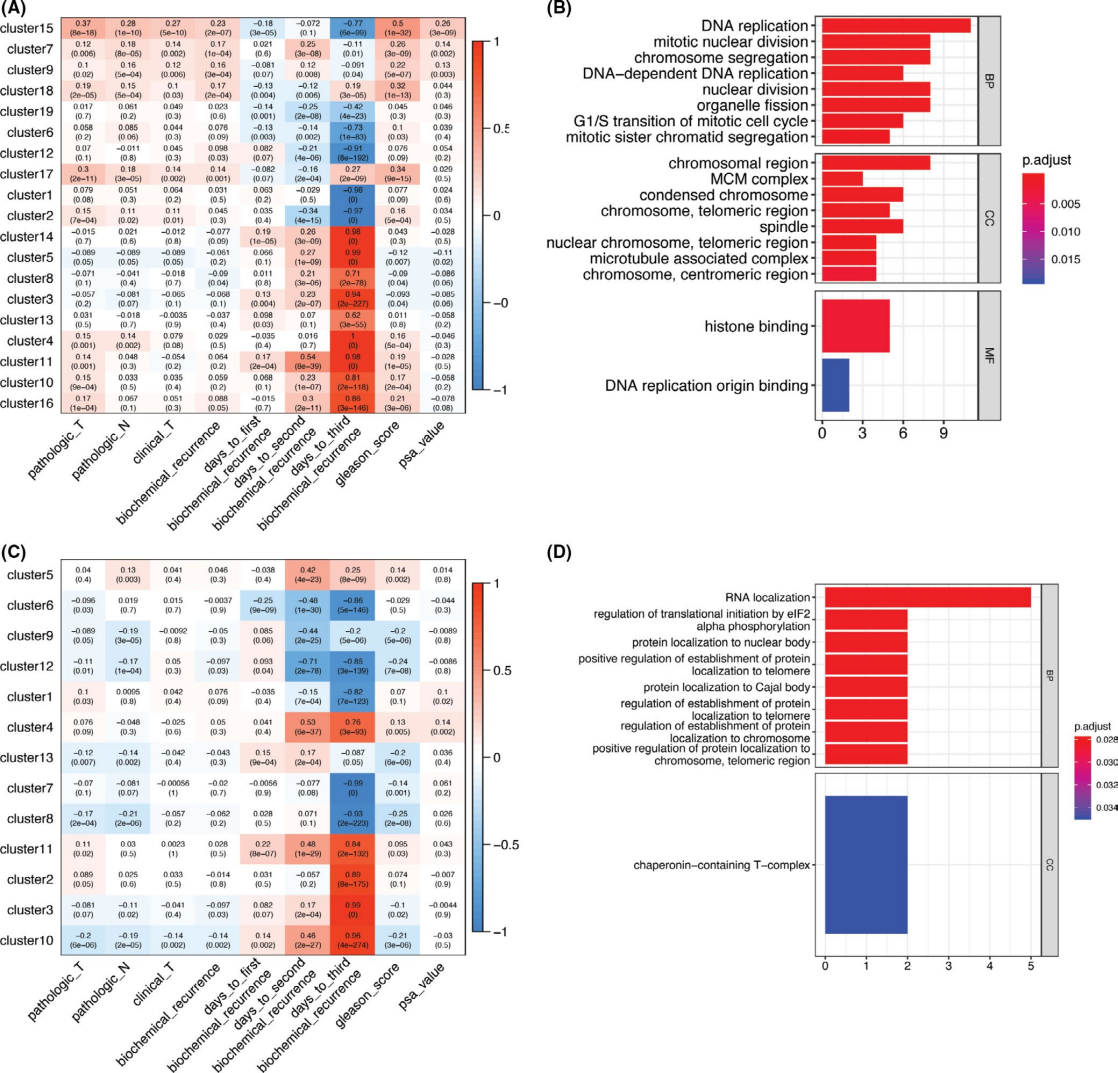
(A) Heatmaps showing the correlation coefficients between the expression modules and selected pathologic and clinical phenotypes. (B) Gene Ontology (GO) enrichment results for the genes in coexpression module 15. (C) Heatmaps showing the correlation coefficients between methylation modules and selected pathologic and clinical phenotypes. (D) GO enrichment results for the genes in comethylation module 10

A schematic diagram depicting the analysis pipeline of this study was shown in Figure 1. We used WGCNA to analyze expression data in 499 PCa samples. We found that 4660 genes could be hierarchically clustered into 20 coexpression modules and that 1305 genes could not be assigned to any modules (Table S1). A main feature of WGCNA is that it can be used to explore the relationship between coexpression modules and phenotypes. We collected clinical information from patients with PCa, including pathologic T and N stages, BCR status, Gleason score, and prostate‐specific antigen (PSA) value, from TCGA. We then used Pearson correlation analysis to assess the correlation between the eigengenes (the weighted average of the expression of all genes in a coexpression module) of each coexpression module and a selected trait (Figure 2A). Notably, patients with higher expression of cluster number 15 genes (which included *STMN1*, *CDCA8*, *RRM2*, *HJURP*, *POC1A*, *MCM2*, *HMGB2*, *TRIP13*, *PTTG1*, *BMP6*, *TCF19*, *KIFC1*, *CBX3*, *RFC2*, *DBF4*, *DNAJC2*, *PBK*, *MCM4*, *MKI67*, *CHEK1*, *LRR1*, *CDKN3*, *C16orf59*, *KPNA2*, *TK1*, *BIRC5*, *CBX2*, *RNASEH2A*, *UBE2C*, *DONSON*, *CHAF1B*, *MCM5*, *and* *KIF4A*) were the most likely to encounter BCR, and the expression levels of these genes negatively correlated with the time of recurrence. Additionally, this cluster’s expression level was positively correlated with pathologic/clinical T stage, pathologic N stage, Gleason score, and PSA value. GO analysis showed that cluster 15 mainly included genes that participate in tumor cell growth and proliferation (Figure 2B). KEGG enrichment analysis showed that this cluster was enriched in DNA replication (adjusted *p*‐value = 1.60e−7) and cell cycle (adjusted *p*‐value = 1.19e−6) pathways.

The methylation pattern of the 4660 genes in the 20 coexpression modules was assessed using WGCNA. Thirteen comethylation modules were identified, and 1781 genes did not form any clusters (Table S2). Correlation analysis between comethylation modules and pathologic traits revealed that hypermethylation of cluster number 10 gene promoters (including *PRPF3*, *PPP2R5A*, *ANGEL2*, *TOMM20*, *SUPT7L*, *YIPF4*, *SOCS5*, *CCT4*, *PTCD3*, *C2orf47*, *NDUFAF3*, *RBM5*, *FAM208A*, *ZBTB11*, *GTPBP8*, *NCBP2*, *YTHDC1*, *TMA16*, *CCT5*, *CNOT6*, *MCUR1*, *NHLRC1*, *EIF2AK1*, *NDUFA4*, *ZSCAN21*, *MEST*, *PAXIP1*, *TNFRSF10A*, *UBE2V2*, *LYPLA1*, *ZNF517*, *CDC26*, *ODF2*, *SURF2*, *CCDC183*‐*AS1*, *NRBF2*, *TBC1D12*, *TDRD1*, *CCDC90B*, *APPL2*, *UTP14C*, *EIF2AK4*, *RPS2*, *B3GNTL1*, *MED25*, *ZNF615*, *COMMD7*, *SNORA60*, *and* *DPM1*) predicted a lower probability of BCR, and the methylation levels positively correlated with recurrence time. Also, the methylation levels of this cluster were negatively correlated with pathologic/clinical T stage, pathologic N stage, and Gleason score (Figure 2C). Hypermethylation of genes in this cluster also GO enrichment analysis showed that the genes in this cluster mainly participate in RNA and protein localization (Figure 2D).

### 3.2 All coexpression modules were associated with expression of at least one 5mC regulator

**FIGURE 3:**
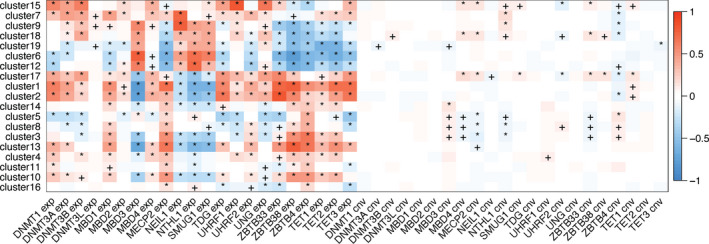
Heatmaps showing the correlations between the expression and copy number variation of 5mC regulators and the coexpression modules identified in PCa, with + and * indicating correlation *p*‐values of <0.05 and <0.01, respectively

To understand the influence of DNA methylation on mRNA expression in PCa, we extracted the expression and copy‐number data of 22 genes that regulate DNA 5mC. These genes include epigenetic writers (DNMTs such as *DNMT1*, *DNMT3A*, *DNMT3B*, and *DNMT3L*), readers (methyl‐CpG‐binding domain proteins such as *MBD1*, *MBD2*, *MBD3*, and *MBD4*, methyl‐CpG‐binding protein 2 [*MECP2*], and other reader genes including *NEIL1*, *NTHL1*, *SMUG1*, *TDG*, *UHRF1*, *UHRF2*, *UNG*, *ZBTB33*, *ZBTB38*, and *ZBTB4*), and erasers (TET family demethylases *TET1*, *TET2*, and *TET3*).^4^, ^26^, ^27^ Correlation analysis identified that all coexpression modules were associated with the expression of at least one 5mC regulator. 5mC regulator expression was more strongly associated with coexpression modules than with corresponding copy number variation (CNV) levels (Figure 3). This demonstrates that 5mC DNA modification has a broad impact on gene expression regulation in PCa.

Analysis of the association between 5mC regulators and comethylation modules (Figure S1) revealed that very few modules were significantly correlated with 5mC regulator expression or CNV. This suggests that 5mC regulators had significantly less influence on promoter DNA methylation than on expression.

### 3.3 Regulation of a coexpression module by its promoter comethylation module

**FIGURE 4:**
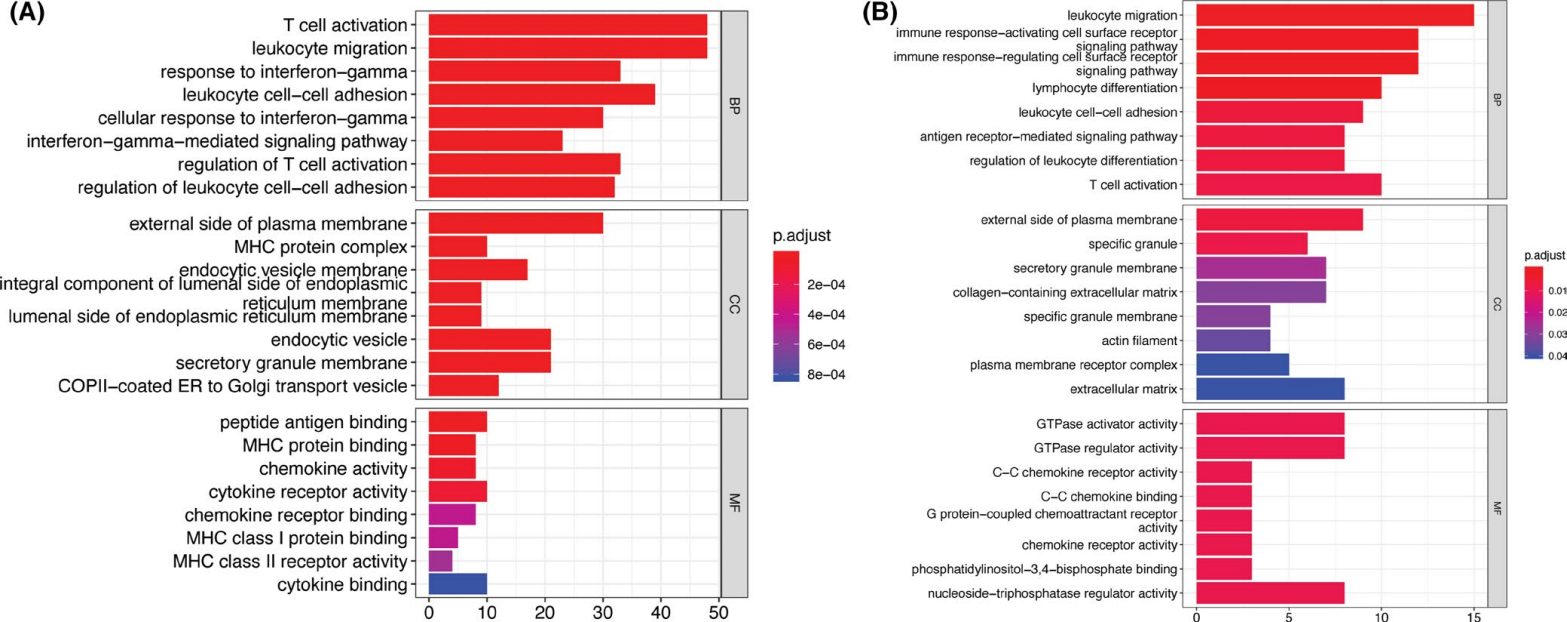
Gene Ontology (GO) enrichment plot for (A) coexpression module 4 and (B) comethylation module 8

To analyze the conservation between coexpression and comethylation networks in PCa, we assessed overlaps between the two datasets (Figure S2A). *Z*‐summary statistics were used to measure conservation scores (Figure S2B,C). Conserved coexpression and comethylation networks were selected based on *Z*‐summary score and cross‐tabulation overlapping *p*‐values. Expression of coexpression cluster 4 was identified as being regulated by its promoter comethylation module, cluster 8. GO enrichment analysis of coexpression cluster 4 (Figure 4A) and comethylation cluster 8 (Figure 4B) showed that these modules were functionally related to the immune response and extracellular matrix activity. Collectively, these results show that promoter methylation levels regulated expressions of genes functionally related to PCa immune response.

### 3.4 DNA methylation regulates the infiltrating immune cells in PCa

**FIGURE 5:**
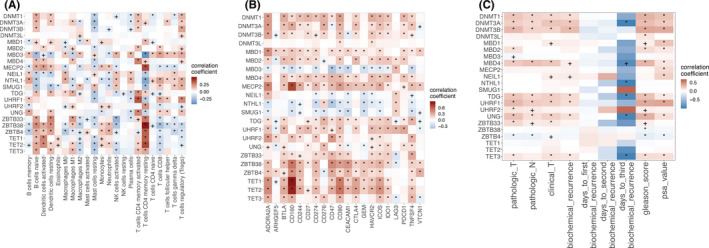
Heatmap of the correlation between the expression of 5mC regulators and (A) tumor‐infiltrating leukocytes (TILs), (B) immune checkpoint blockade (CPB) molecules, and (C) pathologic phenotypes, with + and * indicating correlation *p*‐values of <0.05 and <0.01, respectively

Infiltrating immune cells play vital roles in PCa development and immunotherapy.^28^, ^29^ Therefore, it is important to know if tumor‐infiltrating immune cells are regulated by DNA methylation. CIBERSORT was used to profile 22 classes of tumor‐infiltrating leukocytes (TILs) using PCa RNA‐seq data. The proportion of each immune cell subset was correlated with 5mC regulators using Pearson correlation (Figure 5A). Notably, 5mC regulators were significantly correlated with TILs. CD8^+^ T cells, Tregs, activated NK cells, and M2 macrophages were negatively correlated with 5mC erasers. Naïve B cells, activated CD4^+^ memory T cells, and Tregs positively correlated with 5mC writers. Activated CD4^+^ memory T cells and Tregs showed an overall positive correlation with 5mC readers, while other classes of TILs had diverse association patterns with readers.

We then checked for associations between TILs and coexpression and comethylation modules (Figure S3A,B). Coexpression and methylation modules closely correlated with TILs. Infiltration of naïve B cells and resting CD4^+^ memory T cells was most affected by coexpression modules. Coexpression module clusters 6, 9, 12, 18, and 19 were negatively associated with infiltration and clusters 1, 2, 3, 4, 5, 8, 10, 11, 13, 14, 16, and 17 were positively associated with infiltration. We combined genes with the same association patterns and GO enrichment analysis revealed that genes negatively correlated with naïve B cell and resting CD4^+^ memory T‐cell infiltration were enriched in protein localization. Genes positively correlated with naïve B cell and resting CD4^+^ memory T‐cell infiltration were enriched in extracellular matrix organization. Comethylation modules showed fewer associations with TILs than did the expression modules. Resting CD4^+^ memory T cells were the most affected by the comethylation modules.

### 3.5 Higher 5mC regulator expression marks immunosuppressive microenvironments

Immune checkpoints (ICPs) inhibit immune system activation and play vital roles in immunomodulation. Immune CPB with antibodies targeting cytotoxic T‐lymphocyte antigen‐4 (CTLA‐4) and programmed death‐1/programmed cell death 1 ligand 1 (PD1/PD‐L1) have shown promising results in the treatment of various malignancies.^30^, ^31^

Here, we investigated associations between ICP expression and 5mC regulators (Figure 5B). Twenty‐one ICP genes were downloaded from the HisgAtlas database (http://biokb.ncpsb.org/HisgAtlas/index.php/Home/Browse/). Pairwise Pearson correlations between ICPs and 5mC regulators were calculated for the TCGA PCa data. Most 5mC regulators were significantly positively correlated with ICPs, indicating that the tumor microenvironment in patients with higher 5mC regulator is immunosuppressive (Figure 5B). For example, Tet methylcytosine dioxygenase 1 (*TET1*), Tet methylcytosine dioxygenase 2 (*TET2*), and *MECP2* were strongly associated with *CD160*, an emerging ICP gene whose expression is strongly associated with NK cells and cytolytic CD8 T lymphocytes.^32^

### 3.6 The predictive value of the DNMT3B and DNMT1 writer genes for PCa BCR

Pearson correlation analysis was used to assess the relationships between 5mC regulator expression and pathologic phenotypes (Figure 5C). 5mC writers were positively correlated with pathologic/clinical T stage, pathologic N stage, Gleason score, and PSA value. Moreover, the higher the 5mC writer expression, the greater the probability of BCR.

**FIGURE 6:**
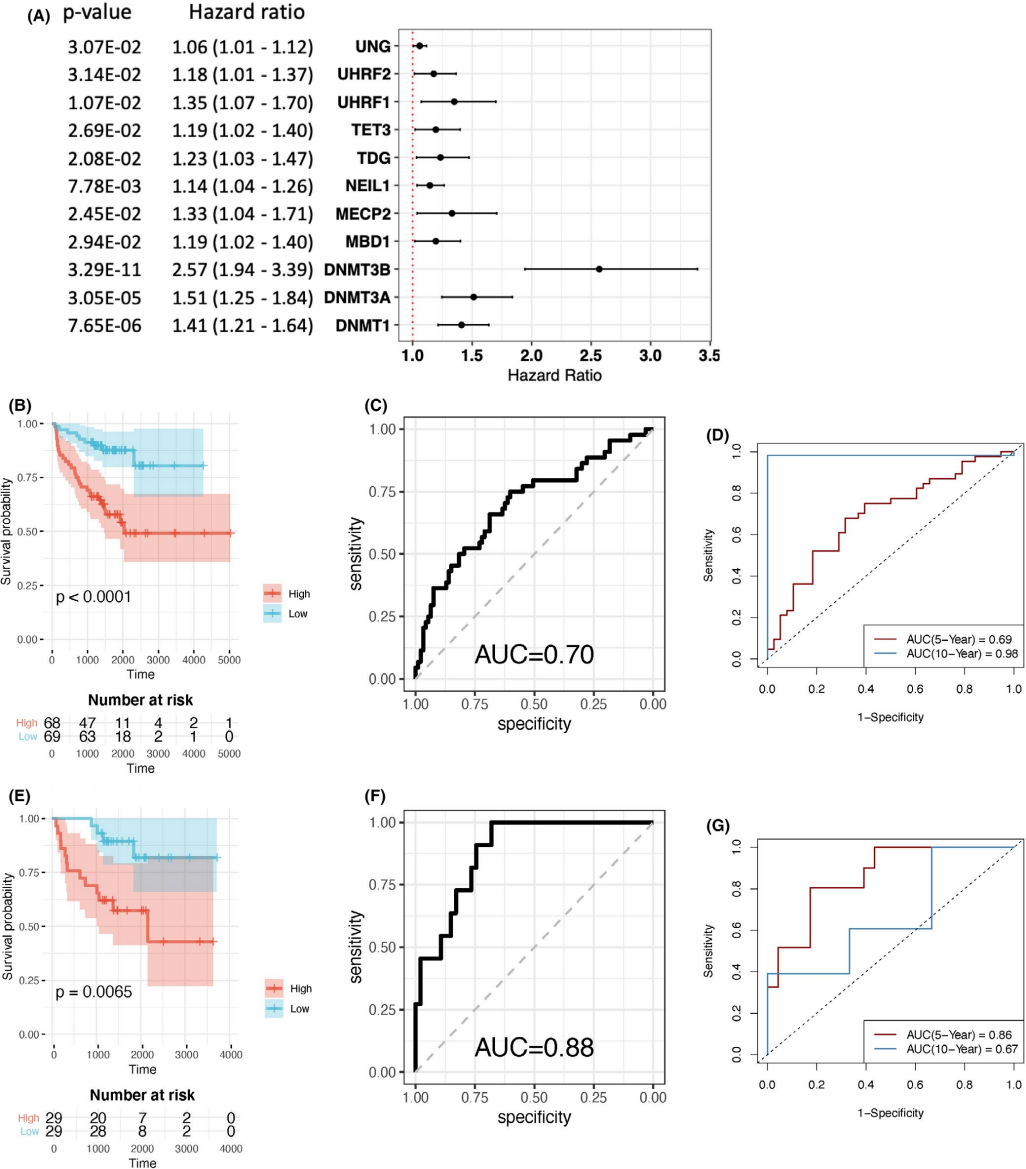
(A) Univariate Cox regression analysis of 5mC DNA methylation regulators in biochemical recurrence (BCR) prediction. (B) Kaplan–Meier survival analysis revealed the significant prognostic value of the DNA methyltransferase (DNMT) risk score in predicting BCR in the training set. (C) Receiver operating characteristic (ROC) curve of the risk score for BCR in the training set. (D) Time‐dependent ROC curve of the risk score for BCR at year 5 and 10 in the training set. (E) Kaplan‐Meier survival analysis revealed significant prognostic value for the DNMT risk score in predicting BCR in the testing set. (F) ROC curve of the risk score for BCR in the testing set. (G) Time‐dependent ROC curve of the risk score for BCR at year 5 and 10 in the testing set

To understand which 5mC methylation regulators contribute most to predicting BCR in PCa, we performed univariate Cox regression analysis using TCGA PCa datasets (Figure 6A). We found that higher expression levels of 11 5mC regulators predicted a greater possibility and earlier time of BCR. Notably, three of the four methylation writers (except *DNMT3L*) significantly predicted BCR for patients with PCa (*DNMT1*: hazard ratio [HR] = 1.41, 95% confidence interval [CI] = 1.21–1.64; *DNMT3A*: HR = 1.51, 95% CI = 1.25–1.84; and *DNMT3B*: HR = 2.57, 95% CI = 1.94–3.39). By LASSO Cox regression algorithm we identified two writer genes, *DNMT3B* and *DNMT1*, and built a risk signature based on the lambda value with minimum CV error in the training set. We determined the score using DNMT3B and DNMT1 expression data the DNMT risk score. Coefficients obtained from the LASSO algorithm were used to calculate the DNMT risk score for the TCGA training dataset. The DNMT risk score formula was:

riskscore=0.48×DNMT3B+0.04×DNMT1

Kaplan–Meier survival analysis was performed in the training group to evaluate the DNMT risk score prognostic value for BCR. A higher DNMT risk score was associated with earlier BCR, with a *p*‐value of 8.25E−05 in the training dataset (Figure 6B). The ROC curve generated from the training datasets achieved an area under the curve (AUC) of 0.70 for BCR occurrence (Figure 6C). The time‐dependent ROC curve is often used to study the diagnostic accuracy of biomarkers on the onset of a disease condition when the disease onset may occur at different times during the follow‐up. Time‐dependent ROC analysis showed AUC of 0.69 at year 5 and 0.98 at year 10, respectively (Figure 6D). The DNMT risk score achieved a *p*‐value of 0.0065 in the Kaplan–Meier analysis (Figure 6E) and an AUC of 0.88 for BCR in the validation dataset (Figure 6F). Time‐dependent ROC analysis showed AUC of 0.86 at year 5 and 0.67 at year 10 in the validation cohort (Figure 6G).

**FIGURE 7:**
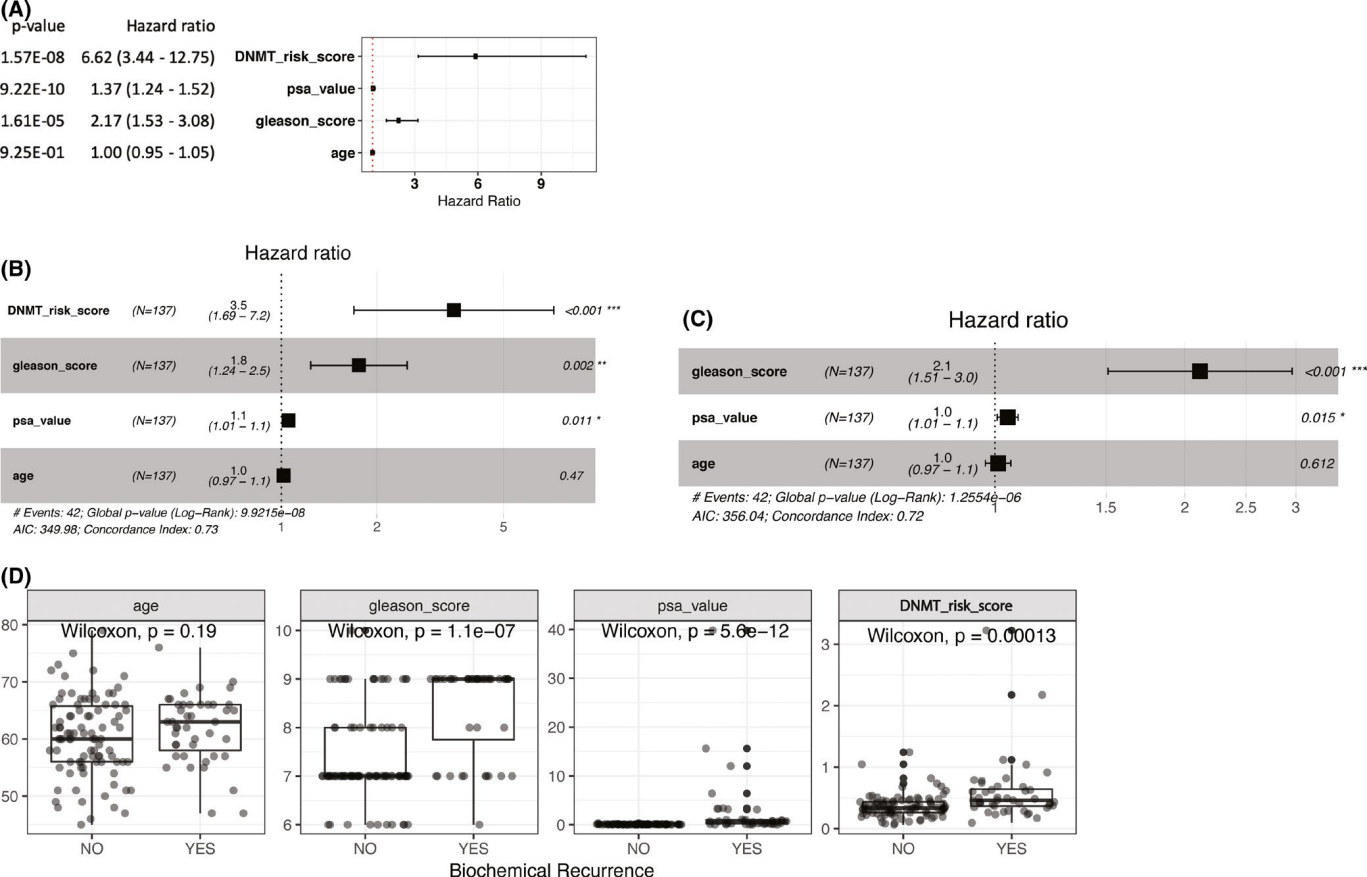
(A) Univariate Cox regression analysis of DNA methyltransferase (DNMT) risk score, prostate‐specific antigen (PSA) value, Gleason score, and age for predicting biochemical recurrence (BCR) in the training set. (B) Multivariate‐adjusted Cox regression analysis of DNMT risk score, PSA value, Gleason score, and age for predicting BCR in the training set. (C). Multivariate‐adjusted Cox regression analysis of PSA value, Gleason score, and age for predicting BCR in the training set. (D) Distribution of age, Gleason score, PSA value, and DNMT risk score across different BCR statuses in the training set

Univariate and multivariate Cox regression analyses were used to evaluate the independent prognostic value of the DNMT risk score for BCR (Figure 7A,B). Univariate analysis results indicated that higher DNMT risk scores were significantly correlated with poor prognosis. Other variables related to earlier BCR included Gleason score and PSA value. Multivariate analysis results showed that a higher DNMT risk score was independently associated with a poorer prognosis in PCa patients including earlier BCR and a higher probability of recurrence. To compare DNMT risk score’s performance with standard factors including PSA value, Gleason score and age, multivariate Cox regression analysis without DNMT risk score were built (Figure 7C). Model performance was improved by adding DNMT risk score to achieve a smaller global log‐rank *p*‐value (9.92e−8 vs. 1.26e−6).

Given the important functions of DNA methylation in tumorigenesis and development, we systematically investigated the relationships between BCR status and factors including DNMT risk score, age, Gleason score, and PSA value (Figure 7D). Wilcoxon testing indicated that the DNMT risk score, Gleason score, and PSA values significantly differed with different BCR statuses in the training set.

**FIGURE 8:**
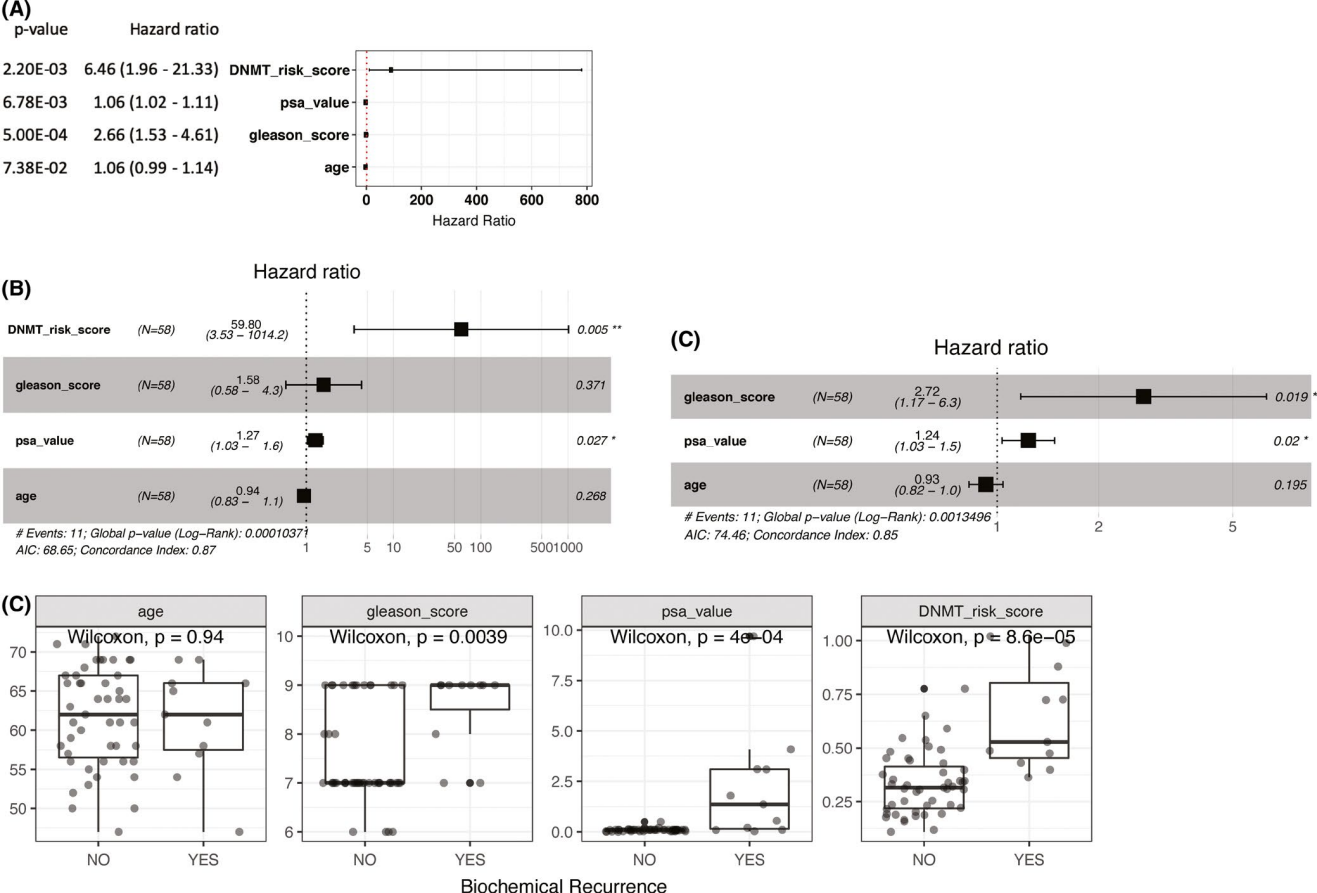
(A) Univariate Cox regression analysis of DNA methyltransferase (DNMT) risk score, prostate‐specific antigen (PSA) value, Gleason score, and age for predicting biochemical recurrence (BCR) in the testing set. (B) Multivariate‐adjusted Cox regression analysis of DNMT risk score, PSA value, Gleason score, and age for predicting BCR in the testing set. (C) Multivariate‐adjusted Cox regression analysis of PSA value, Gleason score, and age for predicting BCR in the testing set. (D) Age, Gleason score, PSA value, and DNMT risk score distributions across different BCR statuses in the testing set

These findings were validated using an independent validation dataset. Results of univariate and multivariate Cox regression analyses in the validation dataset suggested that the DNMT risk score had significant prognostic value (Figure 8A,B). Multivariate Cox regression analysis with DNMT risk score also outperformed than that without (global log‐rank *p*‐value 1.04e−4 vs. 1.35e−3). The DNMT risk score, along with well‐recognized predictors including Gleason score and PSA value, significantly differed between BCR statuses in the validation dataset (Figure 8D). Taken together, these results indicate that features derived from 5mC regulators can serve as independent prognostic factors for BCR and provide supplemental information to classic pathological and biochemical prognostic indictors.

### 3.7 Genes differentially expressed in DNMT risk score‐defined high‐ and low‐risk groups function in different proliferation‐ and metastasis‐related pathways

To analyze whether related pathways were associated with the DNMT risk score, we identified genes that were differentially expressed in the high‐ and low‐risk groups, as defined by the median DNMT risk score (Figure S4A,B). There were 807 genes that were differentially expressed in the high‐ and low‐risk groups (|logFC| > 1, adjusted *p*‐value <0.01). Of these genes, 714 were more highly expressed in the high‐risk group, and 93 were more highly expressed in the low‐risk group. We performed GSEA for the differentially expressed genes (Figure S4C) and found that these genes were mainly related to proliferation and metastasis.

### 3.8 Regulation mechanisms of 5mC regulators in PCa

**FIGURE 9:**
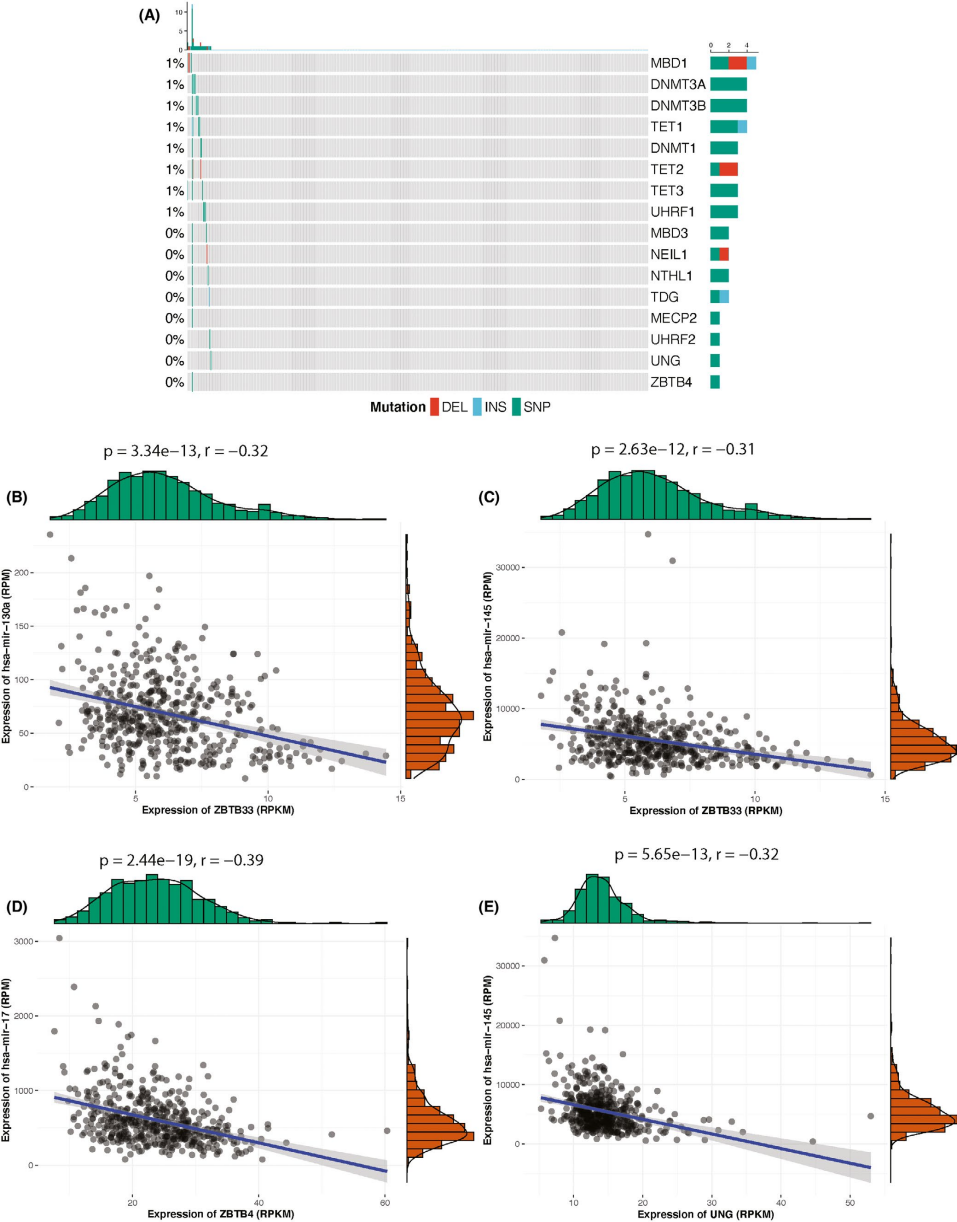
(A) Oncoprint of mutational profiles of the 22 5mC regulators. Scatter plots showing negative associations between (B) hsa‐mir‐130a and ZBTB33, (C) hsa‐mir‐145 and ZBTB33, (D) hsa‐mir‐17 and ZBTB34, (E) hsa‐mir‐145 and UNG

Since expression of 5mC regulators was significantly associated with infiltrating immune cells, ICP activities, and pathologic phenotypes, we thus further investigated regulation mechanisms of 5mC regulators. Mutational landscape indicated 5mC regulators were very conservative during PCa’s tumorigenesis and progression (Figure 9A). miRNAs regulated gene expression post‐transcriptionally by imperfectly binding target mRNAs associated with the multiprotein RNA induced silencing complex (RISC).^33^ By combing both database information and expression profiles, we identified three miRNAs, including hsa‐mir‐130a, hsa‐mir‐145, and hsa‐mir‐17, that showed negative regulation potentials with 5mC readers (ZBTB33, ZBTB4, and UNG, Figure 9B–E).

## 4 DISCUSSION

Previous studies have suggested that changes in nucleotide sequences and epigenetic modifications can confer growth advantage in tumor cells and promote cancer development.^34^ Additionally, the interplay between genetic and epigenetic abnormalities contributes to cancer initiation and progression.

In this study, we conducted a comprehensive analysis to identify the association between DNA methylation and the prognosis of patients with PCa. We used PCa datasets with clinical information, and DNA methylation and expression data from the TCGA. We first identified gene‐methylation pairs by examining coregulation patterns in the PCa samples.

WGCNA of differentially expressed genes identified a cluster of genes whose high expression is associated with several malignant features of PCa. Functional analysis revealed genes in this cluster mainly associated with tumor cell growth and proliferation. This cluster involved minichromosome maintenance (MCM) family members (*MCM2*, *MCM4*, *and* *MCM5*) responsible for DNA synthesis. Recent studies suggested that dysregulated MCMs lead to tumor initiation, progression, and chemoresistance via modulating the cell cycle and DNA replication stress.^35^ *CHECK1* in this cluster is crucial to control speed in the cell cycle. Aberrant expression of *CHECK1* will bring cell proliferation and, eventually, carcinogenesis.^36^ Overexpression of cell‐cycle related genes was demonstrated in various tumor types, including PCa and showed positive correlations with tumor grades. Notably, these genes were associated with an increase in the risk of lethal PCa.^37^ Our analysis suggested upregulation of cell cycle and proliferation‐related genes associated with higher pathologic T stages, clinical T stages, pathologic N stages, Gleason scores, and PSA values. Higher levels of gene expression in this category could also predict earlier BCR and a higher probability of BCR. Furthermore, comethylation analysis also identified a cluster of genes in which hypermethylation predicted a lower probability of BCR. Genes in this cluster predominantly functioned in RNA and protein localization. We identified *CCT4* and *CCT5* in this cluster, which encode a type II chaperonin to fold newly synthesized or misfolded proteins. Previous studies reported that the upregulation of CCT subunits in various cancers and chaperonin is an oncogenic factor.^38^, ^39^ *CCT2* positive breast cancer cells were more invasive and had a higher proliferative index.^40^ Our result suggested that hypermethylation of localization‐related genes may also serve as indicators for better prognosis in PCa.

All coexpression modules were associated with the expression levels of 5mC regulators. However, levels of comethylation modules revealed very few associations with 5mC regulator expression or CNV. This may be explained by the fact that the expression level and methylation level of genes were influenced by complex factors. For example, other forms of epigenetic changes including modification of histone proteins, chemical modification, and chromatin remodeling changes also play vital roles. Another probability is that DNA methylation changes in other regions like gene bodies were also of vital importance. Moen et al. found that tumors with unmethylated *MGMT* promoter and high gene body methylation maintained a high *MGMT* expression, which indicated a positive correlation between gene body methylation levels with its expressions.^41^

Tumor‐infiltrating immune cells play vital roles in PCa development and immunotherapy.^28^, ^29^ Aberrant methylation of immune‐related genes was significantly associated with tumorigenesis and prognostics of various tumor types. Methylation of *LAG3* was strongly correlated with its expression and infiltrations of distinct immune cells in clear cell renal cell carcinoma.^42^ DNA methylation profiling was reported to have the ability to reflect tumor microenvironments and in particular, T lymphocyte infiltrations of the breast cancer.^43^ The above studies suggested possible regulation relationships between methylation modification and immune environments. Therefore, we next focused on analyzing 22 5mC regulators genes and their associations with 22 classes of TILs in PCa. We found that 5mC regulators were significantly correlated with TILs. CD8^+^ T cells, Tregs, activated NK cells, and M2 macrophages were negatively correlated with 5mC erasers. Naïve B cells activated CD4^+^ memory T cells, and Tregs were positively associated with 5mC writers. Activated CD4^+^ memory T cells and Tregs showed an overall positive correlation with 5mC readers. Our findings indicated that besides affecting gene expression profiles of tumor cells, DNA methylation can also impact on tumor immune microenvironment.

BCR is defined as increasing serum PSA levels following radical prostatectomy and is an indicator of disease, including either local recurrence or metastasis to distant sites. Distinguishing patients with high BCR probability are crucial for finding the correct timing to start treatment strategy, thus improving patients’ prognosis.^44^ Previous studies have investigated the potential of DNA methylation signatures as predictors for BCR in PCa patients. High C1orf114 methylation was found significantly associated with BCR, and a three‐gene methylation signature (*AOX1*/*C1orf114*/*HAPLN3*) can predict time to BCR after RP.^45^ A four‐gene LASSO prognostic model (4‐G model) consisting of *APC*, *CRIP3*, *HOXD3*, and *TGFb2* utilizing DNA methylation level was significantly associated with BCR.^46^ These prognostication models suggested that DNA methylation features can serve as a useful tool in risk stratification for BCR. However, previous researches focused on the predictive potentials of methylation levels for selective genesets. Here in this study, we considered the associations between methylation modification and BCR from the perspective of 5mC regulator expressions. We showed that 5mC writers were significantly correlated with clinical phenotypes, including BCR status, and we further built a risk model (the DNMT risk score) for BCR using the *DNMT3B* and *DNMT1* 5mC writers. Compared with traditional methylation markers, measuring expression levels of *DNMT3B* and *DNMT1* are much more convenient and cost‐effective. DNMT activity and protein levels are higher in PCa cell lines than in their nonneoplastic counterparts.^47^ Additionally, DNMT activity is higher in prostatic tissue cultures derived from PCa samples than in those derived from benign prostatic hyperplasia tissue samples and is significantly higher in cultures derived from PCa with Gleason scores ≥7 than in those derived from PCa with Gleason scores <7. Moreover, DNMT activity is higher in PCa cell lines with high tumorigenicity/aggressiveness than in cell lines with low tumorigenicity/aggressiveness.^47^

DNA methylation is a reversible biochemical process and DNMTs have long been investigated as treatment targets for cancers.^48^, ^49^ Decitabine (DRUGBANK ID: DB01262) targets DNMT enzymes, specifically DNMT1, and is indicated for the treatment of patients with myelodysplastic syndromes.^50^ Decitabine has been used in a clinical trial for treating patients with metastatic CRPC (ClinicalTrials.gov). Our results show that higher expression of DNMTs was associated with PCa BCR. This outcome may indicate that DNMT inhibitors have the potential to reduce BCR risk. Besides, our analysis identified broad positive associations between ICPs and DNMTs. Since inhibitors can effectively suppress DNMTs’ expression, our findings implied that DNMT inhibitors might also function in PCa immunotherapy by reducing ICPs expression. Nevertheless, the inferences above still need to be verified by though experiments and clinical trials. As one of the vital epigenetic regulation mechanisms, miRNAs showed reverse associations with several 5mC readers including ZBTB33, ZBTB4, and UNG, which demonstrated overall positive correlations with ICPs. Since miRNAs are also potentially valuable therapeutic targets, miRNA mimic oligonucleotides or constructs reducing the expression of specific miRNAs may also help enhance the effect of immunotherapy.^51^

Most previous cancer analysis regarding methylation was focused on the promoter regions of specific genes. However, few researches focused on the global associations between DNA methylation, gene expressions, and pathologic phenotypes of PCa. Here, we identified regulation relationships from the perspective of coexpression and comethylation modules. Expression patterns of DNA methylation modification‐related enzymes were also profiled. Findings of our research can provide a more comprehensive view and serve as a complement to existing researches. Nevertheless, we limited our study to genes negatively regulated by promoter methylation levels. Since gene expression is affected by complex networks, focusing only on single factors may miss other underlying mechanisms. Also, the size of samples with definite BCR status and time is still limited in the TCGA cohort, which may lead to an unstable outcome of the prediction model. A larger independent PCa cohort with complete follow‐up information was still needed to refine and validate our findings.

## 5 CONCLUSIONS

Here, we describe methylation regulation patterns in PCa at the gene and transcriptome levels. We show that 5mC regulators participate in tumor cell proliferation and regulate the tumor immune microenvironment. 5mC regulator expression is closely associated with PCa clinical phenotypes, including tumor stages, Gleason scores, PSA levels, and BCR. Finally, we built a predictive model using only two features, *DNMT3B* and *DNMT1* expression, and named it the DNMT risk score. This model achieved AUCs for predicting the BCR status of patients with PCa of 0.70 and 0.88 in the training and independent testing datasets, respectively.

## CONFLICTS OF INTEREST

The authors declare no conflict of interest.

## ETHICAL STATEMENTS

No human and animal studies are presented in this manuscript. No potentially identifiable human images or data is presented in this study.

## CODE AVAILABILITY STATEMENT

Codes for this article is available at https://github.com/pyramidsnail/prad‐bcr

## Supporting information

**Figure S1**.
**Figure S2**.
**Figure S3**.
**Figure S4**.
**Table S1**.
**Table S2**.

## Data Availability

Dataset used in this research is publicly available via TCGA GDC Data Portal.
