# Supplementary material for: Expression of 5‐methylcytosine regulators is highly associated with the clinical phenotypes of prostate cancer and DNMTs expression predicts biochemical recurrence
Source: Cancer Med. 2021 Jul 5;10(16):5681–95. doi: 10.1002/cam4.4108 (PMC8366102; doi:10.1002/cam4.4108)
Supplement: Supplementary file 1 — Figure S1. Figure S2. Figure S3. Figure S4. Table S1. Table S2. [file CAM4-10-5681-s001.docx]

Supplementary Material

# Supplementary Figures


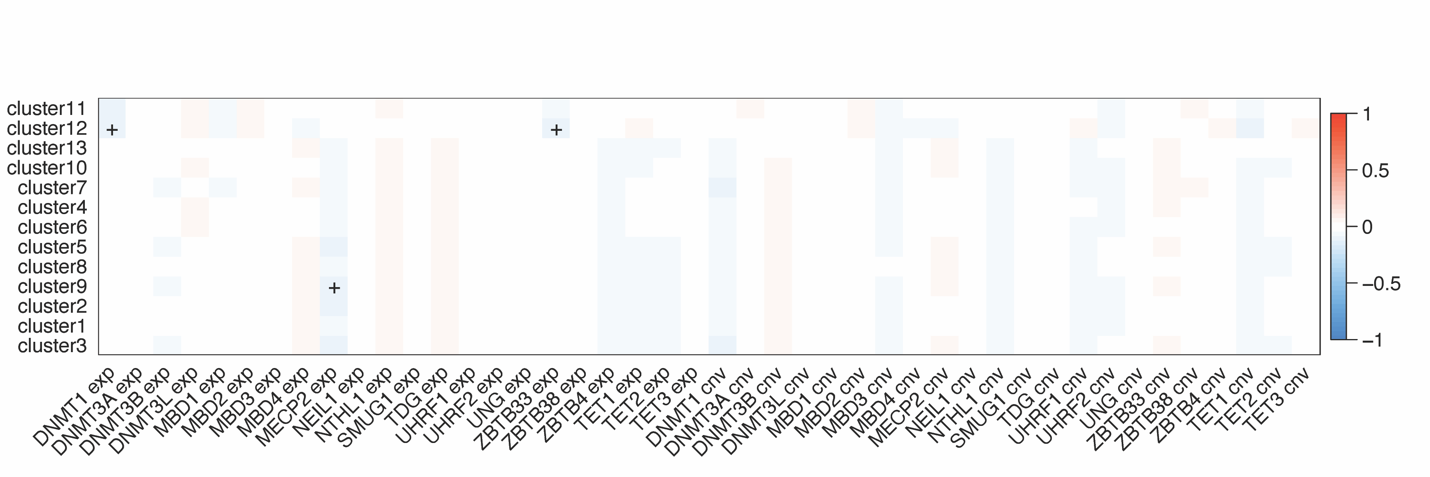


**Supplemental Figure S1**. Heatmaps showing the correlations between the expression and copy number variation of 5mC regulators and the comethylation modules identified in PCa, with + and * indicating correlation p-values of < 0.05 and 0.01, respectively.


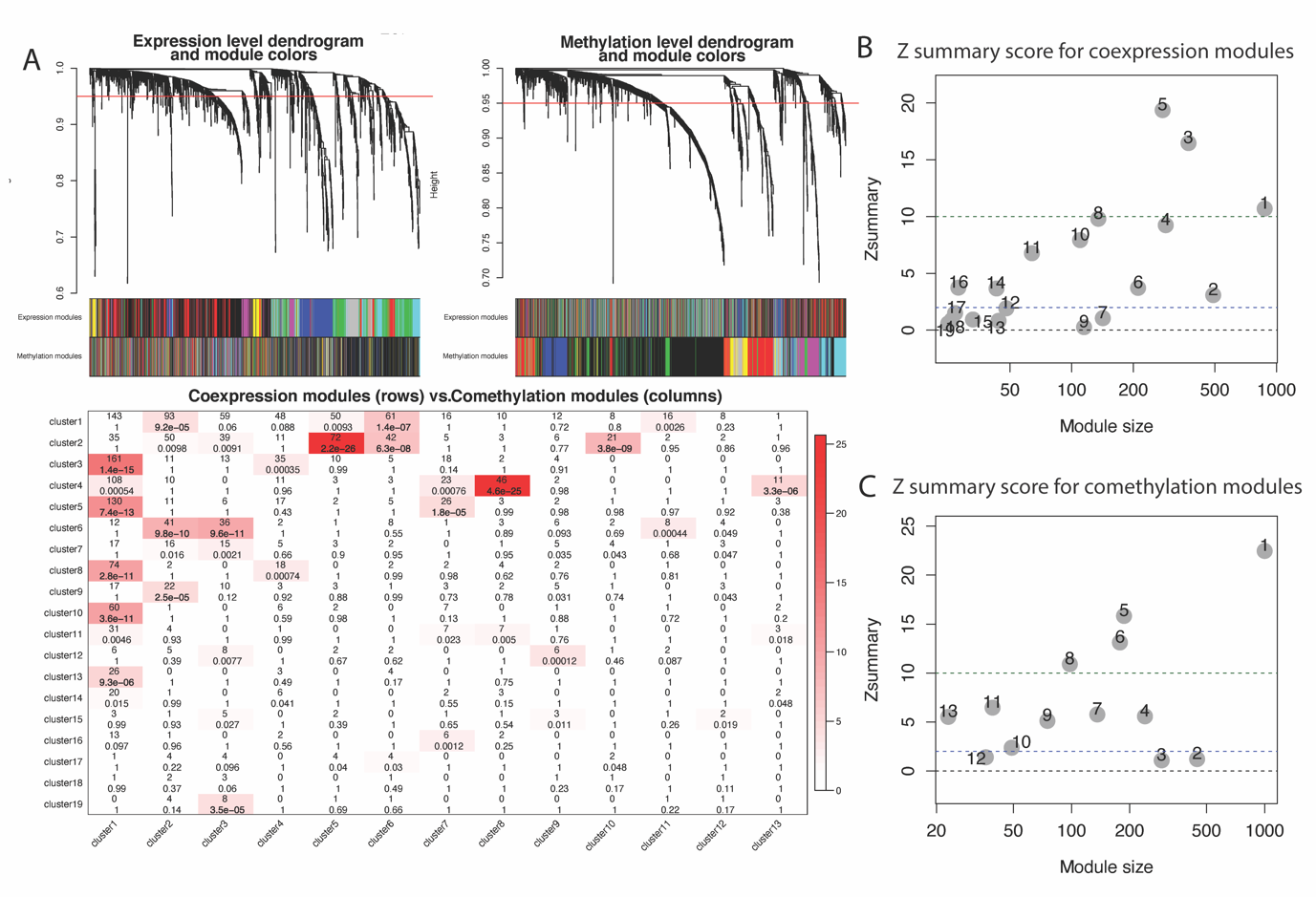


**Supplemental Figure S2.** **(A)** Overlap of the coexpression and comethylation modules with the number of overlapping genes and Fisher’s exact test p-values for observing the overlaps by chance labeled. Z-summary score for each module within **(B)** the coexpression network and **(C)** the comethylation network.


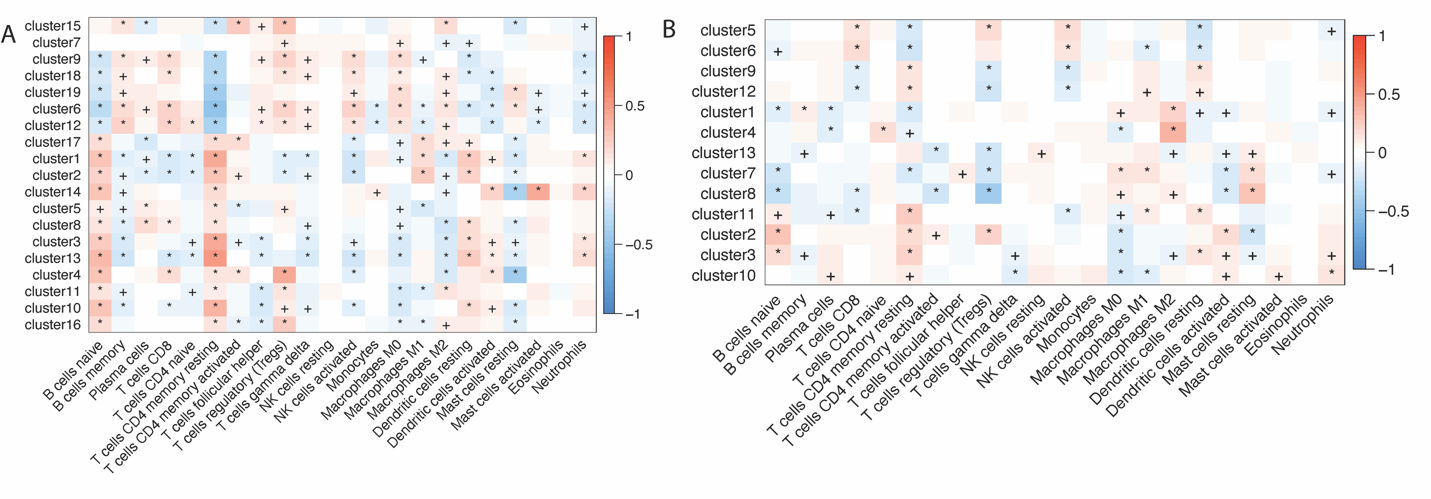


**Supplemental Figure S3**. Correlation heatmaps between the expression of tumor-infiltrating leukocytes (TILs) and **(A)** the coexpression modules and **(B)** the comethylation modules, with + and * indicating correlation p-values of < 0.05 and 0.01, respectively.


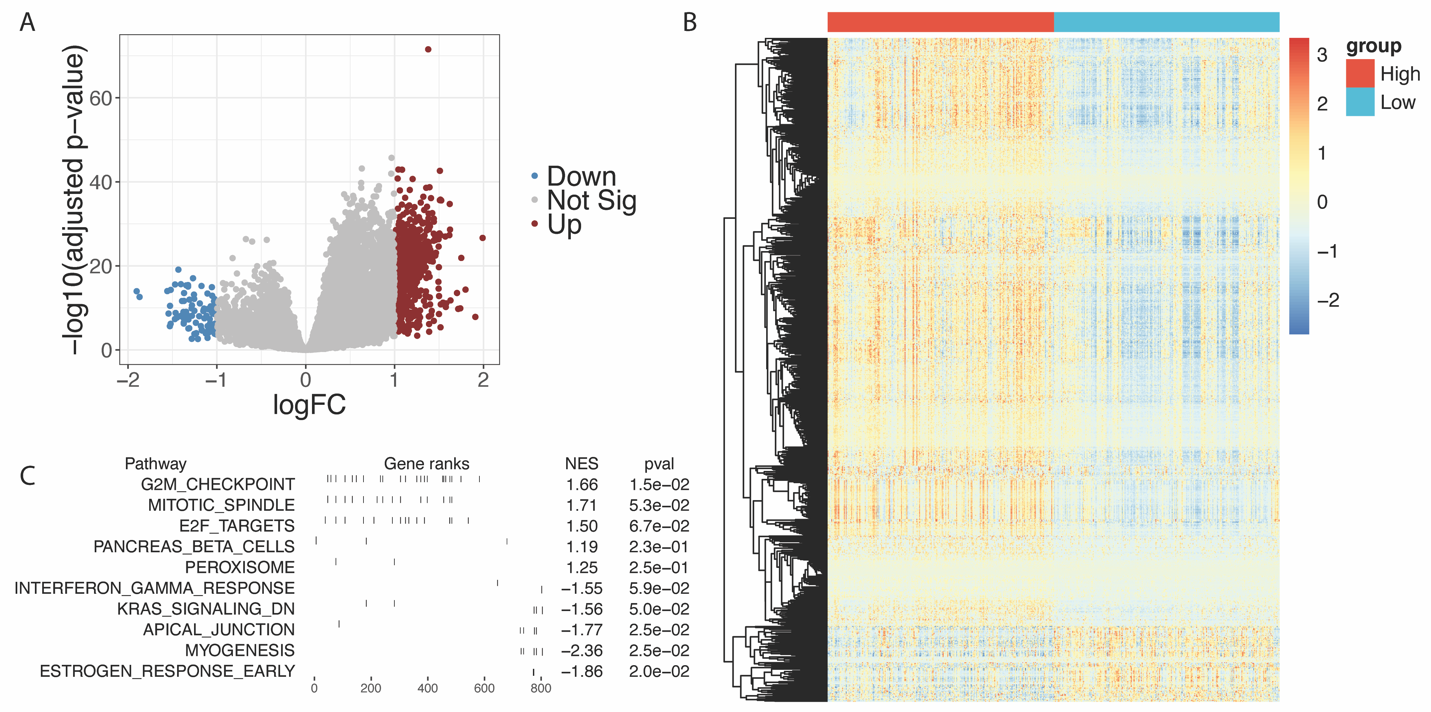


**Supplemental Figure S4.** **(A)** Volcano plot and **(B)** heatmap demonstrating genes differentially expressed between the high- and low-risk groups. **(C)** The top 5 GSEA items for both the positively and negatively enriched pathways in the high-risk group compared to the low-risk group.

# Supplementary Tables

**Supplemental Table S1:** Genes assigned to each co-expression modules

| Cluster | Genes |
| --- | --- |
| Cluster 1 | VWA1; TMEM52; KIF1B; APITD1; DFFA; CASZ1; AGTRAP; PRDM2; NBPF1; MRTO4; MINOS1; HSPG2; ZBTB40; LUZP1; GALE; IFNLR1; ZNF593; CEP85; SYTL1; FAM76A; PHACTR4; KPNA6; ZBTB8OS; THRAP3; EVA1B; LINC01137; C1orf122; AKIRIN1; MACF1; TMEM125; RP1-92O14.3; EIF2B3; FAAH; EPS15; ZCCHC11; RP4-631H13.6; USP24; SLC35D1; TYW3; TTLL7; ZNHIT6; RBMXL1; EVI5; FAM102B; CYB561D1; KCND3; RHOC; RSBN1; VANGL1; PRKAB2; OTUD7B; SETDB1; PIP5K1A; POGZ; OAZ3; RAB13; ADAR; FLAD1; PMF1; ARHGEF11; ETV3; TSTD1; ATF6; C1orf226; ZBTB37; RASAL2; ABL2; SMG7; TRMT1L; DENND1B; ZC3H11A; RP11-739N20.2; PPP1R15B; TMEM81; DSTYK; MFSD4; NUCKS1; DIEXF; HHAT; CAPN2; RP11-504P24.2; ENAH; ITPKB; CDC42BPA; ZNF678; C1orf198; LGALS8; CNST; AHCTF1; ZNF496; AC116614.1; AC144450.1; PXDN; TRAPPC12; KLF11; E2F6; FAM49A; ITSN2; DNMT3A; CAD; KRTCAP3; SUPT7L; EIF2AK2; SOCS5; STON1; B3GNT2; EHBP1; PELI1; PCYOX1; ZNF638; EXOC6B; TTC31; TGOLN2; RNF181; SNRNP200; LONRF2; SNORD89; IL1R1; MRPS9; SOWAHC; MALL; BCL2L11; MAP3K2; IWS1; WDR33; SMPD4; CCNT2; EPC2; RBM43; PKP4; TANC1; BAZ2B; PLA2R1; TTC21B; PPIG; UBR3; AGPS; FAM171B; NAB1; FAM126B; CFLAR; ABI2; INO80D; ZDBF2; AC096772.6; IKZF2; IGFBP2; RNF25; SGPP2; AC104667.3; RAMP1; HDAC4; SNED1; LRRN1; ARPC4; HDAC11; NR2C2; ZNF860; MYD88; ZNF35; ZNF501; LZTFL1; SETD2; PRKAR2A; NDUFAF3; USP4; MON1A; HYAL2; RASSF1; VPRBP; PCBP4; ABHD14B; SELK; ARHGEF3; ZNF717; RP11-159G9.5; NSUN3; NXPE3; PHLDB2; FSTL1; ZXDC; PLXNA1; TMCC1; ZBTB38; TFDP2; GK5; HPS3; B3GALNT1; FNDC3B; PIK3CA; USP13; B3GNT5; PARL; AP2M1; ECE2; TMEM44-AS1; RNF168; ZNF141; RP11-440L14.1; TMEM175; MSX1; EVC; MRFAP1; RP11-539L10.3; AFAP1; ZNF518B; GPR125; SEL1L3; RELL1; N4BP2; RBM47; CENPC; G3BP2; SEPT11; PTPN13; PKD2; PYURF; FAM13A; TET2; METTL14; BBS12; SPATA5; CCRN4L; MGST2; NR3C2; SH3D19; FAM160A1; TRIM2; KIAA0922; RAPGEF2; WWC2; FRG1; EXOC3-AS1; SLC12A7; CTD-2194D22.3; C5orf38; C5orf49; MTRR; C5orf42; AC025171.1; NNT-AS1; NNT; NDUFS4; SNX18; KIF2A; MAST4; POLK; PDE8B; MTX3; TMEM161B; ARRDC3; FAM172A; FER; EPB41L4A; UQCRQ; PURA; PCDHB13; PCDHGB2; PCDHGB5; PCDHGC3; RNF145; PWWP2A; CREBRF; NOP16; CLTB; RP11-423H2.3; ZNF354A; RIPK1; SLC22A23; CDYL; EEF1E1; NEDD9; ATXN1; ALDH5A1; LRRC16A; HMGN4; TRIM26; C6orf136; MDC1; FLOT1; PRRC2A; GPANK1; CSNK2B; ABHD16A; DDAH2; CLIC1; PBX2; BRD2; HSD17B8; SYNGAP1; RPL35P2; SPDEF; DEF6; KCTD20; CCDC167; BYSL; C6orf226; CUL7; PTK7; ZNF318; AARS2; MTO1; CD109; HMGN3; CYB5R4; ZNF292; PNRC1; FRK; TSPYL4; MCM9; HDDC2; ENPP1; RP11-390P2.4; LATS1; IGF2R; RNASET2; MLLT4; NDUFA4; HDAC9; CDCA7L; HOTAIRM1; HOXA6; HOXA13; BLVRA; POLR2J4; ADCY1; EGFR; SNORA22; ABHD11-AS1; CLDN3; WBSCR27; MIR590; GATSL2; WBSCR16; TMEM120A; ZP3; GSAP; CACNA2D1; TP53TG1; LMTK2; BRI3; AC004893.11; TRIM4; ZKSCAN1; RP11-758P17.3; TRIM56; AP1S1; ZNHIT1; SH2B2; NAPEPLD; ATXN7L1; RBM28; TNPO3; MKLN1; SLC35B4; ZC3HAV1L; ZC3HAV1; BRAF; CASP2; CUL1; ZNF746; ATP6V0E2; CDK5; SLC4A2; KMT2C; NOM1; TNFRSF10A; ENTPD4; CTD-2647L4.4; EIF4EBP1; KAT6A; TOX; CYP7B1; PAG1; C8orf59; RBM12B; NIPAL2; COX6C; MTSS1; EFR3A; SLC45A4; JRK; NAPRT; MIR4664; DOCK8; VLDLR; CDC37L1-AS1; ERMP1; ACER2; BAG1; UBAP2; RP11-87H9.2; SMC5; RORB; PRUNE2; TLE1; PHF2; ABCA1; SUSD1; MVB12B; SWI5; ZDHHC12; NUP188; PPP2R4; SURF1; CAMSAP1; LRRC26; DIP2C; FBXO18; UPF2; DCLRE1C; NMT2; PTER; NEBL; COMMD3; KIAA1217; SVIL-AS1; NRP1; RP11-291L22.6; RP11-517P14.2; ZNF32; MAPK8; CSTF2T; BICC1; ARID5B; SGPL1; FUT11; KAT6B; DLG5; POLR3A; TSPAN14; BMPR1A; PAPSS2; LIPA; TNKS2; IDE; TBC1D12; ZNF518A; LCOR; ARHGAP19; EXOSC1; SLC25A28; NDUFB8; MRPL43; KAZALD1; DPCD; LDB1; GBF1; TCF7L2; FAM160B1; ZRANB1; DOCK1; STK32C; FUOM; PAOX; IRF7; PNPLA2; CRACR2B; CD151; TOLLIP-AS1; MRPL17; DENND5A; SBF2; USP47; PLEKHA7; DNAJC24; TRIM44; MADD; PTPMT1; KBTBD4; PTPRJ; CTNND1; SDHAF2; ROM1; UQCC3; TMEM179B; AP000438.2; VEGFB; PPP1R14B; TM7SF2; CDC42EP2; CFL1; BRMS1; CTD-3074O7.5; RHOD; KDM2A; NUDT8; LRP5; MRPL48; RNF169; XRRA1; PAK1; RSF1; NDUFC2; USP35; CEP295; PANX1; CWF19L2; ATM; COLCA1; SIK3; RP11-770J1.3; TRAPPC4; H2AFX; TBCEL; SORL1; SNX19; KDM5A; ERC1; MRPL51; GAPDH; MLF2; PTMS; U47924.27; FOXJ2; DUSP16; CREBL2; ATF7IP; H2AFJ; RASSF8; ITPR2; KLHL42; YAF2; SLC38A2; KRT8; BLOC1S1; WIBG; RPS26; RP11-977G19.5; ZBTB39; DDIT3; MON2; CEP290; EEA1; NDUFA12; GNPTAB; UBE3B; MMAB; MVK; BRAP; ACADS; KDM2B; CLIP1; ZMYM5; N6AMT2; USP12; GTF3A; MTIF3; STARD13; NBEA; SMAD9; PROSER1; NAA16; AKAP11; ZC3H13; LRCH1; RB1; EBPL; UTP14C; UCHL3; STK24; ARHGEF7; MCF2L; TEP1; SUPT16H; DHRS4; IPO4; RABGGTA; ARHGAP5; RALGAPA1; FAM179B; MIS18BP1; KLHDC2; SYNE2; CCDC176; NEK9; NRDE2; CCDC88C; SMEK1; CLMN; GLRX5; RP11-1017G21.5; DYNC1H1; TRAF3; CKB; BAG5; BTBD6; UBE3A; TJP1; KLF13; SLC12A6; BAHD1; CCNDBP1; ADAL; TP53BP1; SERF2; PIGBOS1; PYGO1; RFX7; RNF111; RORA; USP3; PPIB; PIAS1; MYO9A; RP11-817O13.9; HMG20A; PSMA4; ARNT2; HDGFRP3; NMB; PDE8A; AKAP13; POLG; MESP1; ARPIN; ZNF710; HDDC3; FAM174B; LRRK1; JMJD8; HAGH; RP11-715J22.3; RP11-473M20.11; CREBBP; SMIM22; ROGDI; NAGPA; SNN; ABCC1; TMC5; ARHGAP17; NSMCE1; NUPR1; CCDC101; KIF22; CTD-2574D22.4; HSD3B7; ZNF267; LONP2; PAPD5; RP11-44F14.2; RSPRY1; FAM96B; NOL3; ELMO3; ATP6V0D1; ACD; PARD6A; ENKD1; NFATC3; CDH1; VPS4A; NFAT5; PDPR; AP1G1; ZFP1; SYCE1L; CDYL2; GSE1; COX4I1; ZCCHC14; TRAPPC2L; CHMP1A; CENPBD1; RILP; ITGAE; PELP1; SPAG7; RP5-1050D4.5; INCA1; GABARAP; ELP5; MPDU1; TRAPPC1; CTC1; RANGRF; STX8; MAP2K4; UBB; LLGL1; B9D1; ULK2; AKAP10; NATD1; MYO18A; CPD; LRRC37B; WIPF2; RP11-242D8.1; RP11-242D8.2; MAP3K14-AS1; LRRC37A17P; PRAC2; CTD-2377D24.4; ANKRD40; DGKE; MSI2; USP32; TANC2; GNA13; HELZ; LINC00674; ABCA5; ARMC7; SAP30BP; GALK1; JMJD6; TNRC6C; RNF213; BAHCC1; PCYT2; MAFG; ZNF750; RP11-672L10.6; MPPE1; SPIRE1; TTC39C; TAF4B; RNF125; ZNF397; ZSCAN30; MOCOS; SETBP1; ME2; ATP8B1; ZNF532; SOCS6; REEP6; NFIC; MFSD12; ANKRD24; ARHGEF18; NDUFA7; FDX1L; RAVER1; LDLR; SWSAP1; ZNF439; ZNF136; GCDH; NFIX; CTB-55O6.8; TECR; RAB8A; SMIM7; NWD1; LPAR2; ZNF506; ZNF486; ZNF430; ZNF431; ANKRD27; ZNF792; FXYD3; SYNE4; ZNF529; ZNF527; ZNF793-AS1; SIPA1L3; ECH1; ZNF780A; RABAC1; PHLDB3; ETHE1; ZNF234; SLC1A5; ARHGAP35; INAFM1; BCAT2; HSD17B14; FTL; C19orf73; NR1H2; ZNF432; ZNF611; ZNF320; ZNF702P; ZNF415; ZNF761; ZNF331; TFPT; NAT14; ZNF579; ZNF865; EPN1; ZNF582-AS1; ZNF264; ZNF772; ZNF549; ZNF551; ZNF586; ZNF274; ZNF132; RPS10L; PTPRA; PANK2; MCM8; BTBD3; KIF16B; NANP; FRG1B; MCTS2P; NOL4L; COMMD7; ACTL10; CHMP4B; RALY-AS1; NCOA6; MMP24-AS1; SOGA1; RALGAPB; DHX35; SNORD12B; SLC9A8; ATP9A; SON; SLC5A3; DSCR3; C2CD2; ZBTB21; PKNOX1; CSTB; YBEY; TXNRD2; COMT; HSCB; MIR3653; GATSL3; MORC2; DEPDC5; MPST; JOSD1; ATF4; RPS19BP1; RRP7A; POLDIP3; PARVB; PPARA; BRD1; ALG12; STS; SHROOM2; MBTPS2; DDX3X; PCSK1N; PRAF2; KDM5C; ZXDB; LINC01278; TLE1P1; AR; CHIC1; RLIM; PBDC1; ATRX; BRWD3; APOOL; CHM; TRMT2B; GPRASP2; BHLHB9; NGFRAP1; RBM41; MID2; COL4A5; TMEM164; WDR44; IL13RA1; MBNL3; FAM127B; MMGT1; SLC9A6; TXLNGY; KDM5D |
| Cluster 2 | ICMT; RBP7; DDOST; CDC42; SRSF10; CD164L2; STX12; TRIM62; SNIP1; RIMKLA; SLC2A1; ST3GAL3; NDC1; ANKRD13C; PRKACB; LRRC8B; LRRC8D; ZNF326; FNBP1L; SLC35A3; HIAT1; DBT; PRMT6; CLCC1; LRIF1; NRAS; ATP1A1; CHD1L; GOLPH3L; RIT1; COPA; TADA1; DCAF6; BLZF1; MRPS14; STX6; RNF2; CDC73; ZBTB41; ZNF281; KDM5B; YOD1; PPP2R5A; ANGEL2; KCTD3; GPATCH2; EPRS; MIA3; LBR; SDE2; SPRTN; EGLN1; TOMM20; GPR137B; ERO1LB; GPN1; YIPF4; HNRNPLL; FOXN2; MTIF2; PNPT1; PUS10; VPS54; ETAA1; PTCD3; IMMT; MGAT4A; MRPL30; SLC9A2; POLR2D; RAB6C; DARS; ORC4; MMADHC; ARL6IP6; TANK; GORASP2; METTL8; TTC30A; RBM45; GULP1; HNRNPCP2; C2orf69; C2orf47; NIF3L1; CASP8; ACSL3; AP1S3; FBXO36; RAD18; MKRN2; HMGB1P5; DYNC1LI1; RP11-10C24.3; XYLB; KIAA1143; SACM1L; KLHDC8B; CYB561D2; RBM15B; FAM208A; UBA3; PPP4R2; CGGBP1; ARL13B; MINA; ZBTB11; ATG3; GTPBP8; TMEM39A; IQCB1; UMPS; SLC12A8; RAB7A; ATP2C1; CEP63; NCK1; PIK3CB; SLC25A36; HLTF; COMMD2; RNF13; DHX36; SLC33A1; SSR3; MFSD1; MYNN; GPR160; TBL1XR1; ACTL6A; EIF4G1; MAP3K13; LSG1; PPP1R2; PAK2; ZNF595; ZNF721; MFSD7; RP11-529E10.6; LCORL; SLC30A9; BEND4; GNPDA2; COMMD8; YTHDC1; GRSF1; COX18; THAP6; SDAD1; NUP54; SOWAHB; CCNG2; TIGD2; RAP1GDS1; AC004066.3; ARHGEF38; C4orf32; ANXA5; LARP1B; ELMOD2; SMARCA5; ABCE1; PLRG1; MAP9; GUCY1A3; GUCY1B3; FAM198B; RP11-597D13.9; TMA16; SH3RF1; GALNT7; FBXO8; CCT5; GOLPH3; TARS; RAD1; OXCT1; C5orf28; PAIP1; MRPS30; EMB; PARP8; ARL15; GPBP1; ELOVL7; ERCC8; SMIM15; DIMT1; TRAPPC13; NLN; MARVELD2; OCLN; TNPO1; COL4A3BP; POC5; AGGF1; WDR41; SERINC5; DHFR; LYSMD3; TTC37; NUDT12; PGGT1B; CCDC112; SRFBP1; PRRC1; HSPA4; C5orf24; CDC23; SNHG4; PAIP2; RBM27; DCTN4; CYFIP2; CNOT6; MCUR1; HIST1H3E; ZNF322; ZNF184; ATF6B; TBC1D22B; TRAM2-AS1; SMAP1; SLC17A5; SENP6; IBTK; PGM3; UFL1; CCNC; PREP; QRSL1; BEND3; PDSS2; OSTM1; HINT3; MAP7; KIAA1244; TMEM181; C6orf120; FAM20C; TTYH3; CYTH3; FAM220A; MIOS; TWISTNB; HNRNPA2B1; HIBADH; COBL; ZNF107; ZNF138; HSPB1; STEAP4; MTERF1; ANKIB1; BHLHA15; ZCWPW1; SYPL1; CAPZA2; WASL; POT1; GCC1; NUP205; MTPN; RAB19; TCAF1; RP4-584D14.5; PRKAG2; PAXIP1; AGPAT5; CSGALNACT1; C8orf58; PPP2R2A; THAP1; TP53INP1; GRHL2; ZNF252P; AK3; SMU1; HRCT1; HSPB1P1; UBQLN1; C9orf64; NAA35; DAPK1; ZNF189; NIPSNAP3A; RAD23B; C9orf152; PSMD5; STRBP; ODF2; SEC16A; NET1; DHTKD1; PRPF18; FAM188A; BMI1; HNRNPF; ZFAND4; ANK3; NRBF2; REEP3; HNRNPH3; KIAA1279; MCU; P4HA1; C10orf11; TCTN3; CCNJ; PCGF6; TRUB1; TIAL1; C10orf88; BUB3; FAM175B; CTBP2; RP11-45A17.2; RNF141; PDE3B; ARL14EP; FBXO3; API5; EXT2; MTCH2; STX3; DDB1; GANAB; TCIRG1; PRKRIR; PICALM; TMEM135; JRKL; CUL5; DLAT; HYOU1; DPAGT1; APLP2; CDKN1B; WBP11; ASUN; BICD1; DNM1L; TWF1; RP11-446N19.1; TUBA1B; ATF1; CAND1; SLC35E3; YEATS4; ATXN7L3B; METAP2; CDK17; ACTR6; TXNRD1; APPL2; POLR3B; WSB2; DIABLO; CDK2AP1; ZNF664; BRI3BP; ZMYM2; NUPL1; RFC3; KBTBD6; KBTBD7; RCBTB1; DHRS12; NDFIP2; TGDS; GPR180; TFDP1; HNRNPC; G2E3; SNX6; STYX; GNPNAT1; SOCS4; PCNXL4; PPM1A; SIX4; HIF1A; PLEKHH1; SLC39A9; ENTPD5; AREL1; FCF1; SEL1L; PSMC1; TC2N; UBR7; PAPOLA; EIF5; TUBGCP5; FAM98B; EIF2AK4; SNAP23; LCMT2; CASC4; COPS2; ADAM10; ZWILCH; TRAF7; ZNF597; UBN1; USP7; GSPT1; BFAR; IQCK; METTL9; UBFD1; ZNF720; PHKB; TOX3; MT2A; MT1X; CMTM4; TVP23B; PHF12; ZNF207; PSMD11; MSL1; MLX; LSM12; CCDC43; DHX40; RPS6KB1; NT5C; UBE2O; MFSD11; SLC26A11; CSNK1D; WDR45B; CHMP1B; ABHD3; RIOK3; IMPACT; DSC2; DSG2; SLC39A6; TMX3; RP11-162A12.4; PALM; ANO8; ZNF85; ZNF708; RHPN2; UBA2; ZNF146; ZNF568; ZNF233; ZNF229; NUP62; ZNF615; ZNF766; ZNF610; ZNF677; ZNF606; ZNF329; TM9SF4; MAPRE1; DPM1; ZNF217; CABLES2; HSPA13; CXADR; USP16; HMGXB4; ARFGAP3; HCCS; CTPS2; EIF2S3; DYNLT3; BCOR; ATP6AP2; RP2; PPP1R3F; UBQLN2; NAP1L2; MAGT1; TBC1D8B; MORC4; NXT2; AMMECR1; SLC25A43; ZBTB33; SMARCA1; PLXNB3; IRAK1; DNASE1L1; ZFY |
| Cluster 3 | PLEKHG5; VAMP3; SLC25A33; IFFO2; CLIC4; KDF1; AHDC1; TMEM200B; ZMYM6NB; INPP5B; SPATA6; GPX7; CACHD1; WLS; CRYZ; NEXN; FAM69A; PTBP2; SNX7; PALMD; SLC16A4; TSPAN2; SV2A; MTMR11; ANP32E; IL6R; CCT3; ATP1A2; VANGL2; CREG1; ATP1B1; KIAA0040; ANGPTL1; QSOX1; MR1; GLUL; IVNS1ABP; PTGS2; LAD1; ATP2B4; PLXNA2; SERTAD4; TGFB2-AS1; MARC1; HIST3H2A; FAM89A; ID2; LAPTM4A; KCNK3; CGREF1; SRD5A2; ZFP36L2; RHOQ; MEIS1; DYSF; SMYD5; REEP1; KANSL3; RBMS1; CYBRD1; pk; INPP1; SDPR; SPATS2L; AOX1; TNS1; IRS1; PER2; DUSP28; STAC; EIF1B; ENTPD3; ZBTB47; ACOX2; FHIT; ROBO1; STX19; NIT2; BOC; NDUFB4; PODXL2; SEC61A1; SRPRB; AMOTL2; PLSCR4; CPA3; GYG1; MBNL1-AS1; CLDN11; SOX2; C3orf70; MASP1; BDH1; ADD1; CRMP1; WDR1; SGCB; SPATA18; DANCR; RASL11B; KIT; SCD5; PPA2; ARSJ; SYNPO2; FGF2; HHIP; EDNRA; ARHGAP10; PDGFC; GLRB; PALLD; TLR3; ISL1; RNF180; BHMT2; SSBP2; NR2F1; STARD4; MCC; SLC27A6; P4HA2; SH3RF2; MIR143; SMIM3; SLIT3; SH3PXD2B; NEURL1B; MGAT4B; FOXF2; FOXC1; RBM24; ID4; TRIM38; HFE; BTN2A1; HCG11; TRIM27; TEAD3; DST; TPBG; ME1; NT5E; BVES; TRAF3IP2; GJA1; RSPO3; ECHDC1; HEBP2; EPM2A; SLC22A3; ICA1; SP8; JAZF1; WIPF3; MTURN; GLI3; MAGI2; TMEM243; SGCE; PDK4; ZNF655; MDFIC; TES; CAV1; CPED1; CCDC136; FLNC; PPP1R3B; RHOBTB2; TNFRSF10D; FGFR1; PLEKHA2; SFRP1; MRPL15; ASPH; EYA1; MRPS28; C8orf88; SDC2; CPQ; LAPTM4B; ANGPT1; RSPO2; MRPL13; C8orf76; B4GALT1; CNTFR; NPR2; TMEM252; MAMDC2; GNA14; S1PR3; LPAR1; PHF19; STOM; ANGPTL2; SLC27A4; ABO; ITIH5; CAMK1D; PRTFDC1; ALOX5; VSTM4; PRKG1; HK1; CHST3; KCNMA1; LDB3; FAS; PPP1R3C; MYOF; HPSE2; SFXN3; WBP1L; MXI1; AFAP1L2; CHST15; OAT; BCCIP; DCHS1; ST5; MRVI1; DKK3; SPON1; OR7E14P; SAAL1; NELL1; CD44; PAMR1; MEN1; C11orf80; ANO1; PDE2A; SMCO4; PDGFD; MSANTD4; LAYN; SCN4B; VWA5A; ST14; CCND2; C1RL; CLSTN3; DERA; PDZRN4; NCKAP5L; FAIM2; ITGA5; STAT6; WIF1; MSRB3; ANO4; EID3; NUAK1; ACACB; FZD10; SLC46A3; SPG20; TRPC4; DNAJC15; THSD1; EDNRB; SPRY2; MMP14; JPH4; COCH; AP4S1; CFL2; FRMD6; PTGER2; FERMT2; SAMD4A; PLEKHG3; ZFP36L1; PNMA1; TGFB3; CALM1; PRIMA1; ZBTB42; AHNAK2; MEIS2; C15orf52; GNB5; ALDH1A2; CACNA1H; C16orf45; MYH11; PRKCB; ZNF423; LPCAT2; B3GNT9; SLC12A4; MTSS1L; FENDRR; VPS9D1-AS1; DBNDD1; DOC2B; ABR; TLCD2; PITPNM3; MYOCD; MAPK7; DHRS11; AOC3; DUSP3; GJC1; HLF; AXIN2; SYNGR2; EPB41L3; RAB12; B4GALT6; MAPRE2; PSTPIP2; CTIF; ACAA2; RNF152; BCL2; ZNF516; CTC-510F12.2; LPPR2; CLIP3; LRFN1; PNMAL1; TEAD2; MYADM; C20orf194; PRNP; FERMT1; SLC24A3; NINL; TSPY26P; EMILIN3; PTGIS; TIAM1; MN1; RASD2; RP5-1039K5.12; CBX6; CBX7; TEF; MXRA5; MID1; GPM6B; RAI2; SRPX; MAOB; USP11; TFE3; USP27X; NUDT10; ZC4H2; STARD8; NLGN3; SH3BGRL; TMEM35; ARMCX4; ARMCX1; TMSB15A; GPRASP1; RAB9B; ACSL4; CHRDL1; CAPN6; LRCH2; PLS3; KIAA1210; PGRMC1; ELF4; STK26; FLNA |
| Cluster 4 | TNFRSF4; PIK3CD; TNFRSF1B; PADI2; C1QA; C1QC; C1QB; RUNX3; CD52; FGR; THEMIS2; PTAFR; LAPTM5; LCK; IFI44L; IFI44; GBP4; VCAM1; CD53; DENND2D; CHI3L2; CD58; CD2; TNFAIP8L2; S100A9; SLAMF8; CD48; ARHGAP30; FCER1G; FCGR2A; FCGR3A; RCSD1; RGS1; CHI3L1; CDK18; IKBKE; RASSF5; MAPKAPK2; FAIM3; PIGR; RSAD2; PLEK; CD8A; NMI; WIPF1; AC009948.5; SERPINE2; CCL20; SP110; SP140L; SP100; INPP5D; ARL4C; TRANK1; CX3CR1; CCR1; CCR5; UBA7; STAB1; TMEM45A; HCLS1; CD86; PARP9; PARP14; LXN; RARRES1; RTP4; SLC34A2; CXCL6; CXCL1; CXCL10; CXCL13; CCDC109B; SLC1A3; GZMA; IRF1; TCF7; CSF1R; CD74; HAVCR2; LCP2; STK10; SERPINB9; LY86; CMAHP; BTN3A2; BTN2A2; BTN3A3; OR2I1P; HCG4P7; HLA-H; HLA-J; HLA-E; PPP1R18; HLA-C; HCP5; LTB; AIF1; CFB; GPSM3; HLA-DRA; HLA-DQB1; TAP2; PSMB8; PSMB8-AS1; TAP1; HLA-DOA; HLA-DPA1; HLA-DPB1; TAPBP; ETV7; STK38; TREM2; MB21D1; FYN; FAM26F; RAB32; TAGAP; AOAH; ELMO1; IKZF1; FGL2; SAMD9; ARPC1B; GAL3ST4; PILRA; IRF5; TMEM140; TRBV28; GIMAP8; GIMAP7; GIMAP1; TMEM176B; TMEM176A; DOK2; MSC; SLA; AKNA; TRAF1; PTGES; GBGT1; IL15RA; APBB1IP; SRGN; PRF1; SPOCK2; IFIT2; IFIT3; IFIT5; HHEX; ADAM8; IFITM1; LSP1; TRIM22; SAA1; SPI1; MPEG1; SLC15A3; CD5; RARRES3; FERMT3; CCDC88B; CTSW; TBC1D10C; IL18BP; ARAP1; UCP2; CTSC; BIRC3; MMP7; CASP1; IL18; NNMT; CD3E; CD3D; FLI1; CD27; LAG3; DSTNP2; PTPN6; RBP5; C3AR1; CLEC4A; CD69; ARHGDIB; AMIGO2; PCED1B; BIN2; NCKAP1L; GLIPR1; DRAM1; CMKLR1; SELPLG; SH2B3; OAS3; OAS2; N4BP2L1; EPSTI1; PHF11; GPR183; IRF9; GZMB; BATF; GPR68; SERPINA1; WARS; PLCB2; B2M; BCL2A1; HAPLN3; IL32; APOBR; C16orf54; ITGAL; ITGAM; ITGAX; NLRC5; CCL22; MLKL; SLC7A5; CYBA; CXCL16; ZMYND15; CLEC10A; ACAP1; TRPV2; EVI2B; EVI2A; CCL18; LASP1; CCR7; IFI35; FMNL1; ABI3; LIMD2; CD79B; CD300A; RHBDF2; TMC8; LGALS3BP; SLC16A3; CD7; SECTM1; GZMM; EBI3; VAV1; ICAM1; RASAL3; BST2; B3GNT3; JAK3; LRRC25; GMIP; TYROBP; CD79A; APOE; APOC1; PLEKHA4; CD37; NAPSB; NKG7; SIGLEC10; CDC25B; CST7; HCK; PI3; MMP9; CTSZ; RGS19; MX1; PIK3IP1; APOL6; APOL2; APOL1; NCF4; CSF2RB; RAC2; SH3BP1; APOBEC3G; TMSB4X; AP1S2; GPR34; PIM2; VSIG4; IL2RG; SASH3; RENBP; CLIC2 |
| Cluster 5 | SAMD11; ANKRD65; TMEM240; PIK3CD-AS2; HSPB7; UBXN10-AS1; ALPL; ID3; MAP3K6; RCC1; TINAGL1; YRDC; BEND5; GLIS1; GADD45A; LMO4; CNN3; RP4-639F20.1; GSTM4; GSTM2; GSTM5; PROK1; OLFML3; CASQ2; POLR3GL; RP11-196G18.22; S100A6; S100A4; ILF2; SLC27A3; C1orf43; PBXIP1; RUSC1; CASQ1; UAP1; DPT; PHLDA3; LMOD1; G0S2; SERTAD4-AS1; ACP1; MBOAT2; CYS1; OSR1; EMILIN1; RP11-490M8.1; GALM; OXER1; HAAO; SIX2; CNRIP1; VAMP5; MAL; ARID5A; CNOT11; GYPC; LIMS2; RPRM; HOXD13; HOXD9; CYP27A1; DES; SPEG; SLC4A3; TWIST2; GPC1; IL17RE; TIMP4; PLCD1; TMEM158; COL7A1; LAMB2; AMT; SEMA3B; C3orf18; TWF2; PPM1M; GNL3; ABTB1; COPG1; RBP1; CTBP1-AS; WFS1; BDH2; UBE2QL1; SNHG18; CKMT2; TRIM36; PDLIM4; REEP2; FCHSD1; RP11-394O4.5; HMGXB3; GPX3; COL23A1; PXDC1; RP11-420L9.5; APOM; PRRT1; MRAP2; FAM229B; FAM162B; TCP1; GPER1; EIF2AK1; ZNF853; INMT; GNG11; TRIP6; PMPCB; AKR1B1; PTN; ZYX; RARRES2; KCNH2; ACTR3B; MSRA; BMP1; PHYHIP; CLU; SCARA3; RBPMS; PENK; OSR2; LYNX1; LY6E; ZNF34; CCL21; DNAJB5; FAM201A; PGM5-AS1; NINJ1; RP11-535M15.1; PRRX2; PPAPDC3; PTGDS; ZNF485; SNCG; MARVELD1; C10orf2; TNNI2; CDKN1C; PRKCDBP; CYB5R2; AKIP1; RASGRP2; PYGM; LTBP3; EHBP1L1; CD248; RIN1; RP11-867G23.10; MTL5; MRGPRF; LRTOMT; CHRDL2; COLCA2; CRYAB; TAGLN; AP000892.6; FXYD6; VSIG2; ROBO3; FEZ1; ADAMTS8; IFFO1; P3H3; MRPS35; GRASP; IGFBP6; DTX3; ARHGEF25; ALDH2; SERP2; FGF14-AS2; MCF2L-AS1; F10; RNASE4; NDRG2; EFS; REC8; LGALS3; WDR89; RIN3; ASB2; NDN; SCG5; MYZAP; TPM1; FBXL22; RBPMS2; RASL12; C15orf59; CSPG4; FES; TPSB2; HN1L; TNFRSF12A; CDIP1; C16orf89; SBK1; QPRT; CTF1; TGFB1I1; MT1L; MT1A; CCDC102A; RRAD; ZDHHC1; LCAT; WFDC1; SERPINF1; P2RX1; CHRNB1; MYH3; TMEM97; CORO6; MMP28; STAC2; RARA; IGFBP4; PTRF; FZD2; COPZ2; HOXB2; GDPD1; METRNL; MYOM1; TUBB6; CFD; GNG7; PLIN4; ADAMTS10; C19orf66; CNN1; SYDE1; ISYNA1; PLEKHF1; WTIP; PPP1R14A; FBXO17; PRX; NUMBL; CYP2T1P; GRIK5; DMPK; CCDC8; EHD2; HRC; CDC42EP5; PPP1R12C; ADAM33; MYL9; JPH2; SNX21; PLTP; BCAS4; EVA1C; CLIC6; HLCS; PCP4; COL6A2; S100B; CLDN5; EMID1; TBC1D10A; FOXRED2; KCTD17; LGALS1; NDP; RGN; TIMP1; PAGE4; EFNB1; TCEAL2; BEX1; TCEAL7; SMIM10 |
| Cluster 6 | HES4; TNFRSF18; CPSF3L; RP4-758J18.2; RP4-758J18.10; ATAD3A; RER1; THAP3; MECR; ADPRHL2; MRPS15; B4GALT2; CCDC24; RNF220; MAGOH; LAMTOR5; SCNM1; VPS72; JTB; RP11-307C12.12; LAMTOR2; RAB25; RRNAD1; MRPL24; PFDN2; LHX4-AS1; FAM129A; GEMIN6; SNRPG; PCGF1; AUP1; MZT2B; ABCB6; NDUFA10; CAPN10; DTYMK; SEC13; NPRL2; TPRA1; MYL5; CTBP1; OCIAD2; EXOC3; RPL26L1; CTC-338M12.5; ZNRD1; RPP21; MRPS18B; NFKBIL1; LSM2; NELFE; STK19; WDR46; PFDN6; CUTA; RRP36; MRPL2; PRKAR1B; RP11-1275H24.3; C7orf26; MPLKIP; GS1-124K5.4; NSUN5; PDAP1; BUD31; PPP1R35; FASTK; TMUB1; CLTA; ARPC5L; WDR34; SURF2; CACFD1; RABL6; C9orf142; SSNA1; RNF208; RPP38; C10orf35; COMTD1; LZTS2; PWWP2B; SIGIRR; HRAS; RASSF7; NAP1L4; ALKBH3; ARFGAP2; TIMM10; TUT1; EML3; OTUB1; TRPT1; PRDX5; SAC3D1; MAP3K11; DRAP1; CCS; PPP1CA; NDUFV1; LAMTOR1; TIMM8B; RP11-153F5.7; C12orf10; TSFM; FAM109A; MAPKAPK5-AS1; COX6A1; NOC4L; MDP1; TRMT61A; C14orf2; RP11-477I4.4; C14orf79; NUDT14; C14orf80; RPUSD2; TTBK2; NEDD4; SNRNP25; DECR2; PIGQ; RAB40C; FAM195A; FAM173A; HAGHL; RPUSD1; NME3; NUBP2; NDUFB10; SNHG9; RNPS1; AMDHD2; ZNF205; HMOX2; UBALD1; PAGR1; PPP4C; ZNF688; PHKG2; EXOC3L1; EMC6; NEK8; CISD3; SNF8; ICT1; UBALD2; METTL23; C17orf70; CCDC137; ARL16; STRA13; RAC3; RFNG; GPS1; DUS1L; FN3K; MIER2; RNF126; MBD3; ABHD17A; OAZ1; LSM7; SGTA; SIRT6; UBL5; C19orf52; WDR83; STX10; CC2D1A; GIPC1; C19orf60; TBCB; DYRK1B; SNRPA; PPP1R37; ERCC2; SNRPD2; SYMPK; MED25; PPP2R1A; ZNF524; ZNF837; ZNF446; CHMP2A; VPS16; MRPS26; SNHG11; ATP5E; ADRM1; RP5-908M14.9; RBBP8NL; ARFGAP1; SOD1; FAM207A; CECR5-AS1; TRMT2A; MED15; YDJC; SMARCB1; MIF; RNF215; MCAT; SELO; MAPK12; SCO2; TIMM17B; NDUFA1; BCAP31; IDH3G; LAGE3 |
| Cluster 7 | MIR200A; MIR429; RP5-890O3.9; RP11-181G12.2; DFFB; TNFRSF25; IQCC; MTMR9LP; SH3D21; TRIM45; PRPF3; CHTOP; RUSC1-AS1; KIAA0907; RP11-504P24.3; TARBP1; SRSF7; SNORD94; RABL2A; AC093838.4; OGG1; TTLL3; RBM6; RBM5; SNORD19; SNORD69; CFAP44; CCDC14; SDHAP1; PCGF3; SLC26A1; UVSSA; SH3BP2; ANAPC4; BRD9; SDHAP3; TMCO6; ZNF300; C5orf45; TRIM41; ZSCAN9; GABBR1; ATAT1; DDX39B; EHMT2-AS1; EFHC1; SNORD101; SNORD100; COL28A1; AC006042.6; FAM221A; ZMIZ2; SEPT7P2; CCT6P1; RP11-458F8.4; IFRD1; ZNF212; ZNF783; ZNF767P; RP5-855D21.3; SLC25A37; NRBP2; KIFC2; GPT; MRRF; PPP1R26-AS1; CCDC183-AS1; SEC61A2; HSD17B7P2; SEC31B; MTG1; ANO9; AP006621.6; ZNF195; MUS81; ORAOV1; SNORD6; CEP164; POU6F1; PAN2; RP11-347I19.8; SNORA31; UPF3A; LTB4R; SCARNA13; MARK3; PACS2; MTA1; HERC2P2; RP11-540B6.6; GOLGA8A; NEIL1; TIGD7; ZNF75A; PRRT2; GDPD3; RP5-1142A6.9; RP1-59D14.5; EIF4A1; RP11-138I1.3; WSB1; AC010761.8; SUZ12P1; CWC25; LUC7L3; RP11-159D12.2; POLG2; RP13-104F24.2; MYO15B; UNK; SRSF2; ENOSF1; CTD-3128G10.6; FBXL12; ZNF700; MRI1; ARHGEF1; ZNF224; GLTSCR1; PTOV1-AS2; AC018766.4; SNORD88A; ZNF528-AS1; ZNF418; MZF1; ZNF133; SNORA71A; SNORA60; LPIN3; SRSF6; PABPC1L; ZNF335; MIR647; SETD4; POFUT2; HPS4; CTA-984G1.5; SFI1; LINC00899; TUBGCP6; SNORA11; PRKY |
| Cluster 8 | RNF207; FBXO2; RP11-474O21.5; PDPN; TMEM51; EPHA2; ARHGEF19; PAQR7; GJB5; GJB3; EDN2; MOB3C; TTC22; DIRAS3; CSF1; VTCN1; BNIPL; S100A2; S100A16; S100A14; CRABP2; PCP4L1; IER5; ETNK2; CD55; MIR205HG; MIR205; LAMB3; FAM110C; SDC1; CD207; FHL2; TFCP2L1; HOXD10; TMEM237; CCK; TGM4; ALS2CL; CSTA; TF; GPR87; TP63; CLDN1; MUC20; PRDM8; HSD17B13; PITX1; TMEM173; TNIP1; SCGB3A1; SERPINB1; HLA-K; RNF39; GSTA1; DLL1; DEFB1; SOX7; CPA6; ENHO; ROR2; TBC1D2; ASS1; GATA3; MSMB; DKK1; PDLIM1; AVPI1; COL17A1; IFITM10; SCUBE2; DGKZ; GSTP1; MPZL2; WNT5B; SCNN1A; KRT7; KRT5; RARG; NTN4; TRPV4; RASL11A; OLFM4; SLC39A2; AJUBA; NYNRIN; NFKBIA; GPX2; PLEK2; JDP2; DUOXA1; DUOX1; CTD-3094K11.1; PRSS22; CX3CL1; SERPINF2; GGT6; SOX15; NTN1; TNS4; KRT13; KRT15; KRT19; KRT14; KRT17; STAT5A; NGFR; ACSF2; ABCC3; SLC16A5; DSC3; SLC14A1; SERPINB5; RP11-849I19.1; CNN2; CPAMD8; ARRDC2; DMKN; ERF; CXCL17; CD177; KLK1; KLK10; EBF4; CPXM1; SLC4A11; SDC4; WFDC2; CD40; RBM38; BTG3; NEFH; APOBEC3C; APOBEC3F; PLP2; GABRE |
| Cluster 9 | PLEKHN1; RP11-54O7.17; ACAP3; RP4-758J18.13; WRAP73; ZBTB48; FBXO44; CROCCP2; CNKSR1; UBXN11; GPN2; SNORD99; CCDC28B; FAM229A; HYI; MUTYH; AP4B1; PPOX; UCN; ZNF513; MRPL53; STARD7-AS1; WASH2P; BCS1L; TIGD1; HEMK1; MFI2-AS1; RAB24; MXD3; TFAP2A-AS1; PRR3; CCHCR1; EHMT2; DXO; AGER; MIR7111; SNORA33; CTD-2227E11.1; RP1-170O19.17; SNHG15; C7orf43; SRRT; AGAP3; PVT1; RP11-429J17.8; WDR5; LL09NC01-139C3.1; SNAPC4; ANAPC2; EXD3; NSMCE4A; ATHL1; B4GALNT4; LRRC56; LMNTD2; RP11-496I9.1; PIDD1; CTD-2371O3.3; ACCS; PLCB3; ANKRD13D; PUS1; DDX51; OSGEP; METTL17; PABPN1; ABCD4; SNHG10; EVL; SLC25A29; KLC1; RP11-73M18.8; RCCD1; AXIN1; WDR90; RHOT2; CCDC78; CHTF18; TELO2; ZNF598; BRICD5; RP11-304L19.11; RP11-403P17.6; SPIRE2; GPS2; KAT2A; RP11-927P21.4; LLGL2; RECQL5; SIRT7; HEXDC; B3GNTL1; MUM1; DAZAP1; SAFB2; SAFB; PRR22; PCP2; KRI1; EPOR; GTPBP3; TSSK6; YJEFN3; CLASRP; AC074212.6; PTOV1-AS1; SPACA6P; ZNF341; SNHG17; RP11-157P1.4; LIME1; UCKL1; CTA-384D8.36; CHKB; ZRSR2 |
| Cluster 10 | COL8A2; HEYL; PODN; ECM1; CTSK; KIRREL; PEA15; PRRX1; MYOC; RGL1; CFH; NID1; KIF3C; ZEB2; CHN1; FRZB; COL3A1; COL5A2; FN1; IGFBP5; COL6A3; FBLN2; RFTN1; FILIP1L; ABI3BP; HEG1; PDGFRA; LEF1; CFI; NDNF; PCDH10; PCDH18; SFRP2; EGFLAM; CTD-2089N3.1; F2R; EDIL3; PDGFRB; SYNPO; SPARC; ADAM19; EBF1; GFPT2; TNXB; SLC29A1; RCAN2; LAMA4; MAN1A1; LAMA2; MOXD1; TCF21; AKAP12; THBS2; TRIL; TPST1; WNT2; LZTS1; DPYSL2; SULF1; STMN2; MATN2; CTHRC1; EXT1; ASPN; TLR4; MKX; PALD1; CALHM2; ARFIP2; OLFML1; FIBIN; C11orf95; KCNE3; GAB2; PHLDB1; CLMP; PLBD1; ABCC9; CNTN1; ELK3; NFYB; TMEM119; FRY; DZIP1; ITGBL1; KDELC1; COL4A1; NID2; RHOJ; PAPLN; FBN1; FGF7; GAS7; MFAP4; SLFN11; NR1D1; COL1A1; TIMP2; RAB31; SSC5D; SIRPA; RASSF2; ENTPD6; JAM2; TIMP3; CERK; SLC7A3; NAP1L3; SRPX2; DOCK11 |
| Cluster 11 | CDA; FAM110D; GJA4; TIE1; ELTD1; GJA5; PEAR1; CD34; HLX; SEMA3G; KDR; VEGFC; ECSCR; ARAP3; AFAP1L1; NOTCH4; FSCN1; AQP1; RAMP3; STC1; SOX17; HEY1; ENPP2; TEK; SH2D3C; PKN3; MMRN2; LMO2; APLNR; LRRC32; ROBO4; VWF; ACVRL1; NDUFA4L2; PTPRB; RGCC; RNASE1; ADCY4; CLEC14A; CDH5; SCARF1; BCL6B; TMEM88; ARHGEF15; RAMP2; MEOX1; TBX2; ICAM2; DOCK6; NOTCH3; PLVAP; GPR4; RASIP1; HSPA12B; CD93; COX4I2; SOX18; CYYR1; COL18A1; PDGFB; SHANK3; CXorf36; CHST7; ITM2A |
| Cluster 12 | RPL11; RPL5; RPS27; SNRPE; RPS7; AC079250.1; RPS27A; RPL14; TKT; RPL4P4; MRPL1; COX7C; RPS18; TOMM7; STYXL1; PRKAG2-AS1; RPS20; RPL30; TOP1MT; EEF1D; RPL8; SNHG7; RPL27A; C11orf74; RPS3; PHB2; PFDN5; RPL14P1; RPL21; POLR1D; RPS2; NTHL1; RPS15A; RPL26; TOM1L2; RPS2P46; NFE2L1; SNRPD1; RPL17; RPS15; RPS16; SNRPB2; PFDN4; RPL13AP7; RANBP1; RPS4X; RNF113A; RPS4Y1 |
| Cluster 13 | LEPROT; GNG12; SH3GLB1; RAP1A; PTGFRN; TLR5; CRIM1; SEPT10; NFE2L2; KLF7; TMEM43; EIF4E3; ATP10D; SORBS2; SEMA5A; OSMR; ITGA2; IL6ST; MAT2B; FBXO30; SCRN1; SLC39A14; TACC1; TGFBR1; TAF3; CCSER2; HECTD2; SH3PXD2A; YAP1; KDELC2; SACS; KATNAL1; ELF1; LACC1; GLCE; NPTN; ADAMTSL3; NR2F2; MEIS3P1; SLFN5; PRKCA; ITSN1; CELSR1; TBL1X |
| Cluster 14 | ERRFI1; CYR61; MCL1; ADAMTS4; RGS16; ELF3; RP11-510N19.5; BTG2; DUSP2; BHLHE40; BCL6; AREG; DUSP1; IER3; CDKN1A; SERPINE1; GEM; KLF10; NFIL3; KLF4; KLF6; CH25H; DUSP5; ADM; SLC2A3; EMP1; RND1; MMP19; PHLDA1; DUSP6; THBS1; C2CD4B; CCL3; CCL4L1; VMP1; CTD-3252C9.4; ITPKC; PLAUR; THBD; SNAI1; ADAMTS1; LIF; MAFF |
| Cluster 15 | STMN1; CDCA8; RRM2; HJURP; POC1A; MCM2; HMGB2; TRIP13; PTTG1; BMP6; TCF19; KIFC1; CBX3; RFC2; DBF4; DNAJC2; PBK; MCM4; MKI67; CHEK1; LRR1; CDKN3; C16orf59; KPNA2; TK1; BIRC5; CBX2; RNASEH2A; UBE2C; DONSON; CHAF1B; MCM5; KIF4A |
| Cluster 16 | GPR153; COL16A1; HTRA3; IGFBP7; NKD2; AEBP1; IGFBP3; PCOLCE; ENG; VIM; HTRA1; FJX1; SERPING1; LRRN4CL; OAF; MGP; CRIP2; LOXL1; ISLR; HIC1; COMP; TGFB1; RCN3; REM1; SUSD2; GGT5; CSDC2; BGN |
| Cluster 17 | UBE2V2; LYPLA1; TMEM68; IMPAD1; NSMAF; RAB2A; TRAM1; LACTB2; STAU2; UBE2W; TPD52; ZBTB10; CHMP4C; SNX16; WWP1; OSGIN2; PLEKHF2; STK3; POLR2K; ANKRD46; ATP6V1C1; EMC2; UTP23; DERL1; TMEM65; TRMT12; FAM83H-AS1 |
| Cluster 18 | KLHDC9; MTERF3; ENY2; TSNARE1; RP13-582O9.5; ZNF696; RP13-582O9.7; SCRIB; PUF60; GRINA; HSF1; SLC52A2; FBXL6; ADCK5; SLC39A4; AC084125.4; CYHR1; PPP1R16A; MFSD3; LRRC14; C8orf82; ARHGAP39; ZNF251; ZNF517; ZNF7; ZNF16 |

**Supplemental Table S2**: Genes assigned to each co-methylation modules

| Cluster | Genes |
| --- | --- |
| Cluster 1 | RP11-54O7.17; MIR429; ANKRD65; RP4-758J18.7; VWA1; TMEM52; RP4-740C4.5; FAM213B; RNF207; GPR153; HES2; ERRFI1; FBXO2; FBXO44; MTHFR; TNFRSF1B; SNORA59A; TMEM51; HSPB7; EPHA2; UBXN10-AS1; CDA; LINC00339; C1QB; ID3; RPL11; SRSF10; FAM110D; HMGN2; RP1-159A19.4; AHDC1; PTAFR; TMEM200B; TINAGL1; COL16A1; PTP4A2; CCDC28B; MTMR9LP; GJB5; GJB3; GJA4; ZMYM6NB; COL8A2; EVA1B; STK40; INPP5B; MACF1; HEYL; NFYC; CTPS1; EDN2; ELOVL1; HYI; SLC6A9; BEND5; EPS15; KTI12; ZCCHC11; GPX7; PODN; GLIS1; LDLRAD1; TMEM59; USP24; MIR3671; PDE4B; GADD45A; DIRAS3; TYW3; NEXN; IFI44L; ELTD1; SH3GLB1; GBP4; EVI5; RPL5; FAM69A; CNN3; PALMD; VCAM1; NTNG1; HENMT1; CLCC1; GSTM2; GSTM1; GSTM5; CSF1; SLC16A4; LAMTOR5; CHI3L2; RAP1A; AP4B1; ATP1A1; CD58; VTCN1; GJA5; HIST2H2BF; SV2A; MTMR11; ECM1; ANXA9; BNIPL; VPS72; SELENBP1; S100A10; S100A6; S100A4; S100A16; S100A14; SLC27A3; PBXIP1; GPATCH4; CRABP2; PEAR1; VANGL2; ATP1B1; PRRX1; ANGPTL1; ABL2; QSOX1; IVNS1ABP; PTGS2; DENND1B; PHLDA3; RP11-134G8.7; ELF3; CHI3L1; BTG2; ATP2B4; CDK18; MFSD4; IKBKE; RASSF5; CD55; MIR205HG; MIR205; LAMB3; KCTD3; HLX; TLR5; HIST3H2BB; CAPN9; C1orf198; GNG4; NID1; ZNF496; FAM110C; ITGB1BP1; KLF11; FAM49A; KCNS3; SDC1; C2orf43; EMILIN1; FNDC4; RP11-373D23.2; SRD5A2; QPCT; OXER1; HAAO; SIX2; RHOQ; B3GNT2; OTX1; SLC1A4; MEIS1; CNRIP1; DYSF; RAB11FIP5; VAMP5; REEP1; CD8A; MAL; DUSP2; ARID5A; KANSL3; IL1R1; SULT1C4; SEPT10; SOWAHC; MAP3K2; LIMS2; HS6ST1; AC018804.7; ZEB2; ORC4; RBM43; DAPL1; TANK; SP5; DLX2; HAGLR; NFE2L2; PRKRA; FAM171B; COL3A1; NAB1; SDPR; AOX1; CASP8; TMEM237; KLF7; AC096772.6; IGFBP5; TNS1; CYP27A1; FAM134A; DES; SPEG; SGPP2; IRS1; SP140L; SP100; ARL4C; COL6A3; TWIST2; GPC1; PP14571; AC131097.4; CHL1; TMEM43; EIF1B; CCK; ZBTB47; ZNF662; KIAA1143; TMEM158; LZTFL1; COL7A1; SLC26A6; KLHDC8B; USP4; AMT; UBA7; SEMA3F; HYAL2; RASSF1; C3orf18; ALAS1; PPM1M; NISCH; STAB1; NT5DC2; ARHGEF3; ACOX2; EIF4E3; GPR27; FILIP1L; NXPE3; BOC; FSTL1; CD86; CSTA; PARP14; HEG1; PLXNA1; ABTB1; TF; RBP1; PLSCR4; GPR87; RARRES1; MFSD1; TBL1XR1; C3orf70; RPL4P4; MASP1; RTP4; BCL6; PPP1R2; PCGF3; NAT8L; HTRA3; WDR1; ZNF518B; CD38; RELL1; UCHL1; SGCB; RASL11B; PDGFRA; KIT; KDR; IGFBP7; COX18; CXCL6; CXCL1; CXCL10; SEPT11; PRDM8; PRKG2; HSD17B13; NAP1L5; BMPR1B-AS1; TSPAN5; TET2; CFI; ARSJ; NDNF; FGF2; PCDH10; HHIP; ARHGAP10; MAP9; FAM218A; PALLD; WWC2; TLR3; SLC12A7; SEMA5A; ANKH; FAM134B; EGFLAM; OSMR; NNT-AS1; CTD-2089N3.1; GZMA; IL6ST; KIF2A; MAST4; MRPS36; MARVELD2; CARTPT; POC5; PDE8B; BHMT2; SSBP2; PRRC1; P4HA2; PDLIM4; IRF1; PITX1; NME5; PAIP2; TMEM173; PURA; PCDHB3; PCDHB4; PCDHGB5; SPRY4; AFAP1L1; MIR143; RP11-394O4.5; CSF1R; PDGFRB; CD74; SYNPO; GPX3; TNIP1; SPARC; ADAM19; PTTG1; STK10; SH3PXD2B; NEURL1B; GFPT2; SCGB3A1; FOXF2; FOXC1; SERPINB1; SERPINB9; PXDC1; RP11-420L9.5; CDYL; PAK1IP1; ID4; ALDH5A1; HFE; BTN2A1; HCG11; HIST1H2BJ; OR2I1P; HCG4P7; HLA-H; HLA-K; HCG4P5; HLA-J; RNF39; HLA-E; PRR3; PPP1R18; FLOT1; IER3; CCHCR1; TCF19; HLA-C; AIF1; C6orf47; NEU1; CFB; TNXB; PRRT1; NOTCH4; HLA-DOA; HLA-DPA1; PFDN6; SYNGAP1; BAK1; HMGA1; PACSIN1; TEAD3; ETV7; KCTD20; TREM2; CUL9; SLC29A1; EFHC1; CD109; TPBG; ZNF292; BVES; SOBP; OSTM1; MARCKS; FAM162B; GJA1; RSPO3; LAMA2; SNORD100; TCF21; EPM2A; FBXO30; MPC1; FSCN1; ZNF853; SP8; HOXA3; HOXA5; HOXA13; HIBADH; TRIL; WIPF3; SCRN1; AQP1; TRGV10; GLI3; RAMP3; IKZF1; EGFR; MAGI2; SEMA3D; AC002456.2; CDK6; PEG10; PON1; BHLHA15; ARPC1B; FAM200A; ZNF655; TRIP6; TRIM56; SERPINE1; PMPCB; DNAJC2; ATXN7L1; DUS4L; SLC26A4; MDFIC; CPED1; GCC1; CCDC136; IRF5; AKR1B1; TMEM140; PTN; ZC3HAV1; ZYX; CUL1; KRBA1; RARRES2; GIMAP7; GIMAP1; TMEM176B; TMEM176A; ERICH1; DEFB1; PPP1R3B; LZTS1; SLC39A14; RHOBTB2; TNFRSF10D; DPYSL2; CTD-2647L4.4; FGFR1; TACC1; PLEKHA2; PLAT; PENK; FAM110B; CYP7B1; CPA6; MSC; PI15; FABP5; C8orf88; GEM; SDC2; MATN2; ANGPT1; RSPO2; UTP23; ENPP2; MTSS1; SLA; COL22A1; GPT; RPL8; ZNF7; ADAMTSL1; TEK; B4GALT1; AQP3; ENHO; DNAJB5; PGM5-AS1; TMEM252; S1PR3; NINJ1; TGFBR1; ZNF189; ABCA1; KLF4; LPAR1; ORM1; AKNA; PHF19; MRRF; MVB12B; ANGPTL2; TBC1D13; PRRX2; GBGT1; ABO; RXRA; CAMSAP1; ABCA2; UAP1L1; NRARP; KLF6; ITIH5; GATA3; VIM; KIAA1217; APBB1IP; MKX; GPRIN2; RP11-123B3.2; PRKG1; DKK1; BICC1; RHOBTB1; HK1; PALD1; SPOCK2; KCNMA1; CCSER2; MMRN2; SNCG; PAPSS2; CH25H; IFIT2; IFIT3; LCOR; SLIT1; FRAT1; MARVELD1; ZFYVE27; HPSE2; LZTS2; SFXN3; LDB1; SH3PXD2A; COL17A1; DUSP5; PDCD4; AFAP1L2; TIAL1; ADAM8; ATHL1; IFITM1; LMNTD2; PNPLA2; MUC6; KCNQ1; CDKN1C; TRIM68; TRIM22; PRKCDBP; DCHS1; PPFIBP2; ST5; DKK3; SPON1; SAAL1; SAA1; FIBIN; CD44; PAMR1; FJX1; ACCS; DGKZ; SPI1; SERPING1; RP11-142C4.4; STX3; CCDC86; FADS1; EML3; ROM1; LRRN4CL; BSCL2; C11orf95; OTUB1; LTBP3; AP5B1; OVOL1; CTSW; CD248; RIN1; KDM2A; CLCF1; GSTP1; NUDT8; ACY3; MRGPRF; IL18BP; PDE2A; ARAP1; KCNE3; CHRDL2; ARRB1; RPS3; MOGAT2; WNT11; USP35; ME3; YAP1; PDGFD; KDELC2; COLCA2; LAYN; CRYAB; IL18; ZBTB16; ZPR1; TAGLN; CEP164; FXYD6; SCN4B; NLRX1; OAF; CLMP; VSIG2; ROBO4; FEZ1; BARX2; ADAMTS8; ERC1; CCND2; VWF; CD9; PLEKHG6; SCNN1A; LTBR; TAPBPL; USP5; RBP5; SLC2A3; CLEC4A; LOH12CR1; PLBD1; PYROXD1; ABCC9; PDZRN4; YAF2; COL2A1; FAIM2; POU6F1; GRASP; KRT5; RARG; MMP19; WIBG; MSRB3; GLIPR1; DUSP6; ANO4; GNPTAB; DRAM1; NT5DC3; NFYB; NUAK1; TMEM119; ACACB; MYL2; OAS3; TBX3; KDM2B; CLIP1; PUS1; SACS; KATNAL1; KL; RGCC; EPSTI1; DNAJC15; SERP2; GTF2F2; PCDH17; STK24; UBAC2; ITGBL1; METTL17; SLC39A2; NDRG2; MMP14; EFS; JPH4; REC8; LTB4R; ADCY4; NYNRIN; STXBP6; SNX6; SSTR1; CLEC14A; FRMD6; NID2; LGALS3; SYNE2; PLEKHG3; GPX2; RAB15; ZFP36L1; C14orf169; PNMA1; BATF; GPR68; UBR7; PRIMA1; CLMN; SCARNA13; SLC25A29; DYNC1H1; TRAF3; EIF5; AHNAK2; MTA1; SCG5; SLC12A6; MEIS2; C15orf52; BAHD1; PLA2G4D; TTBK2; CCNDBP1; ADAL; B2M; FBN1; FGF7; GNB5; MYZAP; ALDH1A2; RNF111; RORA; C2CD4B; FBXL22; RBPMS2; LRRC49; GRAMD2; PARP6; NPTN; C15orf59; LOXL1; RPP25; CSPG4; HMG20A; ARNT2; NMB; HAPLN3; POLG; ARPIN; HDDC3; NR2F2; CREBBP; CDIP1; NAGPA; C16orf89; CARHSP1; SNN; MYH11; PRKCB; QPRT; PPP4C; CTF1; ITGAX; ZNF423; MT2A; MT1L; MT1M; MT1A; CPNE2; CX3CL1; CCDC102A; GPR56; CDH5; RRAD; NOL3; ZDHHC1; ATP6V0D1; ENKD1; LCAT; SLC12A4; NFATC3; NFAT5; MTSS1L; SLC7A5; CYBA; TRAPPC2L; TLCD2; SERPINF1; HIC1; GGT6; CXCL16; ZMYND15; PITPNM3; CLEC10A; PHF23; ACAP1; CHRNB1; SOX15; AC025335.1; TMEM107; ARHGEF15; NTN1; STX8; GAS7; MYH3; MYOCD; MEIS3P1; MFAP4; UNC119; ALDOC; MYO18A; CPD; SLFN11; CWC25; STAC2; NR1D1; WIPF2; RARA; IGFBP4; TNS4; KRT19; KRT14; KRT17; RAB5C; STAT5A; PTRF; AOC3; IFI35; VAT1; MEOX1; GJC1; FMNL1; PRR15L; NGFR; ACSF2; HLF; MSI2; DYNLL2; TBX2; ACE; LIMD2; ICAM2; RP11-927P21.4; GNA13; AXIN2; PRKCA; CACNG4; ARMC7; MYO15B; GALK1; TMC6; TIMP2; LGALS3BP; MAFG; DUS1L; SLC16A3; SECTM1; ZNF750; DLGAP1-AS1; RAB12; RAB31; APCDD1; ABHD3; TAF4B; RP11-9E17.1; TTR; SLC39A6; SLC14A1; SOCS6; CFD; GNG7; NFIC; CTD-3128G10.6; SH2D3A; VAV1; ARHGEF18; PCP2; ADAMTS10; SWSAP1; CNN1; ACP5; CTD-3252C9.4; RASAL3; RAB8A; F2RL3; CPAMD8; JAK3; LRRC25; ZNF506; PLEKHF1; ANKRD27; UBA2; ZNF599; ZNF30; DMKN; HKR1; PPP1R14A; C19orf33; FBXO17; FBXO27; PRX; CYP2T1P; GRIK5; CXCL17; ZNF229; SNRPD2; DMPK; CCDC8; PNMAL1; RASIP1; PLEKHA4; KLK1; KLK3; KLK12; NKG7; SIGLEC10; SPACA6P; ZNF350-AS1; ZNF611; ZNF761; MYADM; CDC42EP5; PPP1R12C; NAT14; SSC5D; ZNF549; ZNF586; ZSCAN18; ZNF132; ZCCHC3; NRSN2; SDCBP2; SIRPA; CPXM1; SLC4A11; ADAM33; CDC25B; PANK2; PRNP; RASSF2; BTBD3; SLC24A3; THBD; NINL; COX4I2; HCK; TM9SF4; ACSS2; MYL9; SNHG17; EMILIN3; JPH2; SDC4; WFDC2; SNX21; MMP9; CD40; SNAI1; KCNG1; STMN3; SOX18; RGS19; BTG3; RPL13AP7; JAM2; APP; ADAMTS1; TIAM1; CRYZL1; SLC5A3; AP000697.6; PCP4; MIR3197; C2CD2; PKNOX1; COL18A1; COL6A2; S100B; SLC25A18; CLDN5; TXNRD2; SLC7A4; GGT5; MN1; CTA-984G1.5; NEFH; DEPDC5; C22orf42; TIMP3; RASD2; APOL6; APOL2; APOL1; KCTD17; RAC2; LGALS1; JOSD1; APOBEC3C; APOBEC3F; APOBEC3G; FAM83F; TEF; CSDC2; MCAT; FAM118A; CERK; SHANK3; MXRA5; STS; GPM6B; TMEM27; SMS; ARX; SRPX; MAOB; RGN; TIMP1; PRICKLE3; PAGE4; IQSEC2; MAGED2; STARD8; SLC7A3; HMGN5; SRPX2; TMEM35; ARMCX1; GPRASP1; GPRASP2; TCEAL7; NGFRAP1; COL4A5; LRCH2; DOCK11; KIAA1210; PGRMC1; SEPT6; SMARCA1; ELF4; SMIM10; MMGT1; GABRE; BCAP31; FLNA; PRKY |
| Cluster 2 | ACAP3; RP4-758J18.2; RP11-345P4.9; THAP3; PIK3CD; CASZ1; AKR7A2; KDM1A; CNKSR1; KDF1; SYTL1; CD164L2; IQCC; NCDN; YRDC; C1orf210; CCDC24; FAAH; PRMT6; OAZ3; RAB13; RP11-307C12.12; SLC50A1; RUSC1-AS1; ASH1L-AS1; KIAA0907; RAB25; TMEM79; CCT3; RRNAD1; IGSF9; TSTD1; PVRL4; PFDN2; PPOX; FCER1G; MGST3; DPT; PRDX6; RFWD2; LAD1; RP11-465N4.4; SNRPE; C1orf116; YOD1; ARV1; GNPAT; LGALS8; CNST; RNASEH1-AS1; RSAD2; ZNF513; KRTCAP3; XDH; STON1; PUS10; NFU1; MCEE; CNOT11; MRPS9; MALL; PSD4; POLR2D; DARS; PHOSPHO2; ACSL3; AC104667.3; NDUFA10; SNED1; ANO7; OGG1; SLC6A11; PPARG; THRB; RP11-10C24.3; PLXNB1; MON1A; HEMK1; PRKCD; STX19; MINA; PHLDB2; TIMMDC1; SEC61A1; GATA2; COPG1; EFCAB12; ATP2C1; CEP63; TM4SF1-AS1; LIPH; ZNF721; MYL5; RP11-440L14.1; TMEM175; DGKQ; SPON2; CTBP1-AS; TMEM128; CCDC149; SLC34A2; RBM47; OCIAD2; RASSF6; SOWAHB; CCNG2; MRPL1; AC004066.3; ARHGEF38; LARP1B; SFRP2; LINC01207; GALNT7; FAM149A; EXOC3-AS1; SLC6A19; C5orf38; C5orf49; DNAH5; FAM105A; CTD-2139B15.1; PARP8; PART1; SMIM15; OCLN; WDR41; FAM172A; SHROOM1; TMCO6; ZBED8; RNF144B; TRI-TAT2-2; PPP1R10; C6orf136; NFKBIL1; SLC44A4; SKIV2L; ATF6B; TAP1; SPDEF; DEF6; FKBP5; C6orf132; GNMT; RRP36; CUL7; MRPL2; HMGN3; FIG4; AMD1; FRK; SNORA33; MAP7; RP3-325F22.5; IL20RA; PACRG; PRKAR1B; LFNG; CYTH3; ICA1; CTD-2227E11.1; CLDN3; TMEM120A; STYXL1; HSPB1; CACNA2D1; TP53TG1; CROT; DBF4; BRI3; PDAP1; ZKSCAN1; SRRT; IFT22; WASL; C7orf73; RAB19; TRPV6; ZNF767P; ATP6V0E2; CTA-398F10.2; MSRA; ASAH1; SH2D4A; CLU; ATP6V1H; IMPAD1; LACTB2; CHMP4C; RNU6-925P; TP53INP1; KB-1562D12.1; GRHL2; RP13-582O9.7; MIR4664; FAM83H-AS1; SCRIB; KIFC2; ZNF34; TLE1; C9orf64; C9orf152; ENG; SURF1; WDR5; LL09NC01-139C3.1; SEC16A; RPP38; NEBL; C10orf107; C10orf35; ANKRD22; IDE; BLNK; MORN4; C10orf2; DPCD; C10orf95; TMEM180; NSMCE4A; STK32C; SIGIRR; HRAS; RP11-496I9.1; RNF141; KCNJ11; LDHA; LUZP2; CTD-2589M5.4; MS4A8; RP11-855O10.2; TUT1; UQCC3; CHRM1; AP000438.2; PPP1R14B; RP11-867G23.8; C11orf80; SSH3; PPP1CA; RP11-660L16.2; LRTOMT; MRPL48; LRRC32; NDUFC2; TMEM135; COLCA1; CD3E; SNORD14E; SLC37A2; TMEM45B; APLP2; ST14; NCAPD3; GLB1L2; MRPL51; MLF2; U47924.31; PTPN6; U47924.27; LYRM5; ERGIC2; ACVRL1; KRT8; RP11-153F5.7; ERBB3; AGAP2-AS1; TSPAN31; PTPRB; PHLDA1; SLC25A3; BTBD11; RP11-347I19.8; ZNF664; NOC4L; IFT88; MIPEP; GTF3A; MTIF3; SPG20; UPF3A; OSGEP; HOMEZ; RP6-65G23.3; PPP1R13B; PACS2; SNURF; SPINT1; PLA2G4F; HMGN2P46; PYGO1; ADAM10; BBS4; LMAN1L; RP11-817O13.9; RP11-81A1.6; FAM103A1; FURIN; TMEM8A; PIGQ; RAB40C; FAM195A; WDR90; RHOT2; JMJD8; HAGHL; NTHL1; BRICD5; RNPS1; ZG16B; PRSS22; ZNF205; HMOX2; UBN1; USP7; GSPT1; TMC5; NUPR1; PHKG2; HSD3B7; PRSS8; CCL22; MMP15; GOT2; CMTM4; FAM96B; EXOC3L1; CDH1; AP1G1; GSE1; COX4I1; CHMP1A; GAS8; RILP; SERPINF2; INCA1; DVL2; GABARAP; GPS2; TNK1; TRAPPC1; RANGRF; SCO1; PLD6; B9D1; DHRS13; DHRS11; SRCIN1; CISD3; AC087491.2; GRB7; JUP; RAMP2; WNK4; CNTD1; CCDC43; MAP3K14-AS1; EPN3; MKS1; GDPD1; USP32; CYB561; C17orf58; LLGL2; UNK; EVPL; UBE2O; ST6GALNAC1; BAIAP2-AS1; SLC38A10; PCYT2; FN3K; RIOK3; TTC39C; DSC2; ZNF397; MALT1; PALM; CIRBP-AS1; DAZAP1; LSM7; TINCR; SAFB; NDUFA7; RAVER1; LDLR; EPOR; RGL3; ASNA1; GIPC1; CYP4F11; MED26; NWD1; ANO8; LPAR2; C19orf40; FXYD3; SYNE4; ECH1; CEACAM20; CBLC; APOC1; PPP1R37; AC074212.6; SULT2B1; FAM83E; C19orf73; AC018766.4; PPP2R1A; ZNF415; SLC52A3; RPS10L; CST7; APMAP; BPIFB2; NNAT; ZNF335; SLC13A3; CDH26; CABLES2; RBBP8NL; DNAJC5; CLDN8; TFF3; TFF1; FAM207A; POFUT2; RANBP1; HPS4; SEC14L2; INPP5J; MPST; TTLL1; PARVB; LINC00899; TTC38; MAPK8IP2; REPS2; MID1IP1; RBM3; KDM5C; AR; JPX; TMSB15A; RNF128; PLXNB3; IDH3G |
| Cluster 3 | CPSF3L; RER1; PEX10; C1orf174; FBXO6; MRTO4; DDOST; LDLRAP1; ZNF593; CEP85; RPS6KA1; MECR; LINC01137; CDCA8; RP1-92O14.3; RNF220; TTC39A; RP11-191G24.1; DHCR24; SAMD13; OLFML3; RP1-178F15.4; CHTOP; SCAMP3; COPA; USP21; UAP1; TADA1; RP11-739N20.2; TMEM206; MARC1; ZNF678; NTPCR; TARBP1; LINC01341; CGREF1; CAD; MTIF2; RNF181; MRPL30; LONRF2; CHST10; ACOXL; TMEM177; MZT2B; PTPN18; DPP4; METTL8; RBM45; HJURP; DTYMK; IL5RA; ARPC4; PRKAR2A; RBM6; NPRL2; RBM15B; POC1A; SNORD19; SNORD69; FAM3D; FHIT; NIT2; UMPS; SLC12A8; PIK3CB; GPR160; TMEM41A; DANCR; STAP1; PPA2; MGST2; CLGN; ABCE1; RP11-597D13.9; GOLPH3; TARS; CTD-2517O10.6; PPAP2A; RNF138P1; HEXB; MTX3; DHFR; TRIM36; SPATA24; RPL26L1; NOP16; TRIM41; TRIM26; RPP21; DDX39B; DXO; STK19; RPS18; WDR46; ZBTB22; FOXP4; BYSL; AARS2; CYP39A1; C6orf57; SMAP1; SLC17A5; PGM3; CGA; PREP; SMPDL3A; KIAA1244; TTYH3; FAM220A; PSPH; ASL; ABHD11-AS1; ZP3; STEAP4; LMTK2; BUD31; RP11-758P17.3; AP1S1; PLOD3; SLC35B4; CDK5; AGAP3; MRPS28; TPD52; WWP1; RBM12B; RPL30; NIPAL2; STK3; COX6C; ANKRD46; NCALD; DEPTOR; C8orf76; HSF1; LRRC14; ZNF251; CLTA; TOMM5; NAA35; FBP1; ARPC5L; SLC27A4; ZDHHC12; MED22; CACFD1; PPP1R26; SNAPC4; SNHG7; C9orf142; LRRC26; ANAPC2; SSNA1; EXD3; CDNF; ZNF485; MCU; NDUFB8; MRPL43; GBF1; CRACR2B; TOLLIP-AS1; OR7E14P; FOLH1; AP001258.4; TMEM179B; TM7SF2; MAP3K11; NDUFV1; MTL5; SNORD6; TMEM218; PHB2; WBP11; HOXC6; PA2G4; APOF; TSFM; POLR3B; FAM109A; BRAP; PSMD9; EIF2B1; RFC3; NBEA; KBTBD7; CARKD; MDP1; LRR1; KLHDC2; PPM1A; MNAT1; NRDE2; TTC7B; CCDC88C; SERPINA11; SNHG10; GLRX5; GABRG3; PHGR1; SNAP23; SERF2; PPIB; ZWILCH; RP11-279F6.1; LINGO1; PSMA4; MESDC1; ZSCAN2; CHTF18; LA16c-312E8.4; TELO2; NME3; NUBP2; SNHG9; TRAF7; AMDHD2; TXNDC11; AC004381.6; UBFD1; SEPHS2; SLC38A7; TXNL4B; CDYL2; DBNDD1; GLOD4; PELP1; RP5-1050D4.5; ALOX12-AS1; AC010761.8; NEK8; ERBB2; MAPT; LUC7L3; POLG2; METTL23; SYNGR2; TK1; ARL16; RAC3; OGFOD3; ENOSF1; IMPA2; SNRPD1; TMEM241; PCAT18; C18orf32; RNF126; RPS15; SGTA; SIRT6; ANKRD24; LPPR2; PRKCSH; GCDH; FARSA; SMIM7; MIR3189; TMEM161A; TSSK6; ZNF146; SUPT5H; ZNF404; GLTSCR1; LMTK3; SNORD88A; TMC4; ZNF579; ZNF542P; ZNF274; MZF1; SNRPB2; NCOA6; SNHG11; SNORD12B; PMEPA1; HLCS; ADARB1; YBEY; CECR5-AS1; SEPT5; HMGXB4; ARFGAP3; BRD1; ZRSR2; GRPR; TIMM17B; GJB1; IL13RA1; SLC25A5; RNF113A; LDOC1 |
| Cluster 4 | WRAP73; ZBTB48; RBP7; DHRS3; RP11-474O21.5; CAMK2N1; IFNLR1; THEMIS2; BMP8B; PGM1; CYR61; LRRC8B; F3; TSPAN2; MEX3A; ETV3; PCP4L1; KIAA0040; RGS16; LAMC2; RNF2; LMOD1; ETNK2; G0S2; SERTAD4-AS1; SUSD4; ENAH; CDC42BPA; FAM89A; GPR137B; ID2; TRIB2; OSR1; KCNK3; RP11-490M8.1; CRIM1; EIF2AK2; ZFP36L2; FAHD2CP; FAHD2B; CD8BP; TFCP2L1; TANC1; RBMS1; CYBRD1; WIPF1; CHN1; ZNF385B; INPP1; ABI2; IHH; SERPINE2; BOK; LRRN1; BHLHE40; TIMP4; TRANK1; ENTPD3; CDCP1; TMEM45A; AGTR1; HPS3; B3GALNT1; SPTSSB; CLDN11; B3GNT5; MAP6D1; CHRD; ZNF141; ADD1; ANAPC4; N4BP2; AREG; PKD2; FAM13A; TIGD2; CCDC109B; SH3D19; FAM160A1; SORBS2; NKD2; NNT; ARL15; LOX; AC009014.3; GABBR1; XXbac-BPG248L24.10; APOM; BRD2; PIM1; RCAN2; SENP6; NT5E; ECHDC1; SLC22A3; DACT2; SMOC2; CTA-293F17.1; AC004540.5; MTURN; POLR2J4; ORAI2; CAV1; FLNC; ZNF467; KCNH2; ANGPT2; SOX7; SCARA3; EIF4EBP1; TSPYL5; KLF10; TRAPPC9; GSDMD; VLDLR; CNTFR; PRUNE2; NFIL3; ROR2; PHF2; TBC1D2; PTGR1; SUSD1; TLR4; RALGDS; PPP1R26-AS1; IL15RA; CAMK1D; ALOX5; MAPK8; SRGN; H2AFY2; CHST3; KAT6B; DLG5; FAS; HHEX; MMS19; LOXL4; SCD; PPAPDC1A; ANO9; NAP1L4; CYB5R2; SBF2; SLC35C1; MADD; PTPRJ; VEGFB; LRP5; SIK3; WNT5B; MGP; ARHGDIB; KLHL42; BICD1; NCKAP5L; WIF1; NTN4; ELK3; TRPV4; WSB2; FRY; LRCH1; ESD; TRIM13; THSD1; MCF2L-AS1; PCK2; PTGER2; PAPLN; ACOT4; JDP2; FAM189A1; KLF13; RP11-758N13.1; DUOXA1; DUOX1; TPM1; HEXA; KIF7; ZNF710; CACNA1H; B3GNT9; HSD11B2; ZNF232; SLC16A13; CENPV; TMEM98; OSBPL7; COPZ2; ABCC3; HELZ; LINC00674; SLC16A5; METRNL; COLEC12; EMILIN2; B4GALT6; CTIF; RNF152; GZMM; ZBTB7A; ZNF846; ZNF439; ZNF69; PRKACA; SYDE1; GTPBP3; ARRDC2; ISYNA1; ZNF486; RHPN2; CTC-526N19.1; ZNF792; SCN1B; SIPA1L3; CNTD2; ARHGEF1; LYPD3; ZNF836; EBF4; FERMT1; LAMP5; NOL4L; PLTP; ATP9A; ZNF217; IFNGR2; TRPM2; KDELR3; PPARA; CELSR1; MAPK12; DDX3X; CHST7; USP11; PLP2; NLGN3; TTTY14 |
| Cluster 5 | VAMP3; KIF1B; WLS; FNBP1L; RBM8A; CHD1L; GOLPH3L; TNFAIP8L2; JTB; ATF6; DCAF6; RGL1; TRMT1L; ZNF281; GPATCH2; RPS7; ETAA1; C2orf42; SNORD94; STARD7-AS1; SLC9A2; CCNT2; EPC2; PKP4; C2orf69; INO80D; IKZF2; RNF25; TCAIM; SACM1L; FOXP1; CGGBP1; ARL13B; TMEM39A; NDUFB4; MBNL1-AS1; SLC33A1; MYNN; FNDC3B; LSG1; GNPDA2; CENPC; OSTC; METTL14; BBS12; SMARCA5; PLRG1; MSMO1; FBXO8; SDHAP3; C5orf28; ESM1; SETD9; GPBP1; DIMT1; TRAPPC13; NLN; TAF9; TNPO1; COL4A3BP; POLK; ATG10; XRCC4; COX7C; ARRDC3; TTC37; CCDC112; SRFBP1; C5orf24; SH3RF2; RBM27; RAB24; SLC22A23; TMEM14C; HIST1H3E; TRIM27; PRRC2A; PPIL1; CYB5R4; PNRC1; UFL1; CCNC; QRSL1; MAN1A1; LATS1; C6orf120; CBX3; JAZF1; SNHG15; TMEM248; ZNHIT1; BCAP29; POT1; MTPN; INTS9; RDH10; STAU2; UBE2W; ZBTB10; POLR2K; MYC; SMC5; NIPSNAP3A; NET1; PRPF18; COMMD3; CSTF2T; ARID5B; FAM35A; TNKS2; TCTN3; ARHGAP19; WBP1L; FAM160B1; MKI67; TSG101; ARL14EP; TRIM44; API5; EXT2; XRRA1; JRKL; BIRC3; KBTBD3; CWF19L2; FOXJ2; CDKN1B; MRPS35; TWF1; OS9; CAND1; SLC35E3; ATXN7L3B; CEP290; METAP2; RNF10; BRI3BP; KBTBD6; TSC22D1; ZC3H13; SUGT1; TGDS; G2E3; AP4S1; FAM179B; STYX; PCNXL4; WDR89; CCDC176; PSMC1; FAM98B; AKAP13; CCDC101; PAPD5; CHD3; TVP23B; ULK2; C17orf75; ZNF207; AC005336.4; ZNF780A; MAPRE1; SRSF6; HSPA13; USP16; TAB1; ATXN10; LMF2; AP1S2; MBTPS2; DYNLT3; UBQLN2; FAAH2; ZXDB; LINC01278; NAP1L2; ATRX; BRWD3; SH3BGRL; CHM; WBP5; TBC1D8B; RBM41; MID2; ZBTB33; ZFY; KDM5D |
| Cluster 6 | PRDM2; LUZP1; ZCCHC17; PARS2; LRRC8D; HIAT1; DBT; KCND3; VANGL1; PTGFRN; C1orf43; RP11-312J18.5; CREG1; ZBTB37; SMG7; PPP1R15B; NUCKS1; RP11-504P24.2; HNRNPLL; AC007318.5; SNORD89; RAB6C; ARL6IP6; TTC21B; PPIG; NIF3L1; CFLAR; FBXO36; NR2C2; UBA3; VGLL3; NSUN3; IFT122; DHX36; PIK3CA; GRPEL1; SLC30A9; COMMD8; G3BP2; SDAD1; NUP54; SPATA5; NR3C2; NDUFS4; LYSMD3; CDC23; FCHSD1; CREBRF; CTC-338M12.5; ATXN1; HIST1H2BG; BTN2A2; BTN3A3; HIST1H3H; HCP5; CLIC1; LSM2; RPL35P2; MTO1; POPDC3; HINT3; TCP1; MLLT4; DLL1; GPER1; RP11-121A8.1; MPLKIP; ZNF107; MTERF1; CAPZA2; TNPO3; NUP205; BRAF; AGPAT5; PPP3CC; TMEM68; NSMAF; RAB2A; SLCO5A1; EMC2; ENY2; ZNF252P; UBQLN1; SWI5; TAF3; DHTKD1; KIAA1279; PCBD1; TAF5; MTG1; AMPD3; DNAJC24; ARFGAP2; CTNND1; RP11-794G24.1; PRDX5; EIF1AD; LAMTOR1; RP11-864N7.2; RNF169; RSF1; PANX1; CUL5; FDX1; HYOU1; RNF26; MAGOHB; DUSP16; ATF7IP; ASUN; MON2; RPL14P1; EEA1; CDK17; ACTR6; TXNRD1; PROSER1; ELF1; TFDP1; AJUBA; ARHGAP5; RALGAPA1; SAMD4A; ABCD4; AREL1; FCF1; BAG5; UBE3A; IVD; RMDN3; SLC27A2; RFX7; PDE8A; KIF22; DHODH; ZNRF1; GABARAPL2; CYB5D2; UBB; NATD1; MLX; RP11-242D8.1; CHMP1B; TPGS2; SETBP1; ATP5A1; BCL2; LINC00909; DIRAS1; MLLT1; PRDX2; NFIX; ZNF431; ZNF527; LRFN1; ZNF432; SLC9A8; PFDN4; SON; ITSN1; SRSF9P1; MIF; HSCB; TBC1D10A; SFI1; RAI2; EIF2S3; ATP6AP2; RP2; PORCN; RLIM; APOOL; TRMT2B; MORC4; PRPS1; WDR44; NDUFA1; IRAK1 |
| Cluster 7 | TNFRSF4; IFFO2; HSPG2; CD52; FGR; TIE1; SLC16A1; CTSK; ATP1A2; CASQ1; ADAMTS4; PIGR; CAPN2; SDE2; CYS1; GPN1; PKDCC; RTN4; FHL2; BCL2L11; HOXD13; HOXD9; FRZB; IL17RE; CCR1; HCLS1; SOX2; MFSD7; CCRN4L; EDNRA; UBE2QL1; F2R; GLRX; TCF7; ECSCR; SMIM3; SNORA74B; RBM24; MDC1; PBX2; LRRC73; VEGFA; AKAP12; RP1-170O19.17; EVX1; INMT; AEBP1; GATSL2; PCOLCE; NOM1; DEFA6; PHYHIP; STC1; SFRP1; OSR2; EXT1; HRCT1; MAMDC2; STOM; PPAPDC3; PTGDS; DIP2C; NMT2; LDB3; AVPI1; ASCL2; OLFML1; MRVI1; SLC15A3; CD5; RPLP0P2; FERMT3; PYGM; ANKRD13D; TBX10; AP000892.6; PHLDB1; CD27; IFFO1; LAG3; C1RL; CLSTN3; RASSF8; SLC38A2; OR7E47P; IGFBP6; ITGA5; STAT6; NDUFA4L2; ARHGEF25; STARD13; GPR183; F10; RNASE1; PAX9; CRIP2; ISLR; TNFRSF12A; TGFB1I1; TOX3; NLRC5; CKLF; WFDC1; SCARF1; ITGAE; P2RX1; BCL6B; TRPV2; LLGL1; AKAP10; CORO6; LASP1; DUSP3; HOXB2; HOXB7; ABI3; CUEDC1; CD300A; CBX2; CCDC137; TUBB6; TYROBP; CLIP3; RINL; TGFB1; HRC; MAFB; CLIC6; MX1; NCF4; CSF2RB; SH3BP1; RP5-1039K5.12; MAFF; CHKB; BGN |
| Cluster 8 | TNFRSF25; PADI2; ALPL; C1QA; C1QC; RUNX3; UBXN11; LAPTM5; LCK; RP4-631H13.6; FAM102B; CD2; TPM3; ARHGAP30; RCSD1; RGS1; DSTYK; FAIM3; CD34; RRM2; ITSN2; PLEK; SP110; INPP5D; CX3CR1; CCR5; ALS2CL; ABHD10; TPRA1; CPA3; ARAP3; RGS14; NEDD9; FAM65B; XXbac-BPG248L24.12; LTB; PSMB8; PSMB8-AS1; CDKN1A; ZNF318; SYTL3; MYO1G; PILRA; SH2B2; DOK2; BMP1; SH2D3C; PRF1; HTRA1; DOCK1; IRF7; IFITM10; OR51E1; RARRES3; CCDC88B; TBC1D10C; ANO1; NNMT; CD3D; C3AR1; NCKAP1L; SELPLG; DIABLO; OLFM4; SIX4; TC2N; PLCB2; DECR2; IL32; APOBR; C16orf54; ITGAL; MYO1C; EVI2A; SLFN5; CCR7; CD79B; SEPT9; TMC8; BAHCC1; CD7; KRI1; BST2; COMP; CD79A; PLAUR; HSPA12B; CD93; BCAS4; RBM38; CTSZ; LIF; PDGFB; SCO2; GPR34; CXorf36; IL2RG; SASH3 |
| Cluster 9 | ANGPTL7; PLEKHM2; MRPS15; IPO13; RBMXL1; ILF2; LHX4-AS1; TMEM81; AGT; TGOLN2; COL5A2; GNL3; SPCS1; CFAP44; ACTL6A; USP13; TMEM44-AS1; SDHAP1; C1QTNF3; SNX18; UQCRQ; ZNRD1; MRPS18B; ABHD16A; TOMM7; HOTAIRM1; ZMIZ2; SLC26A3; RPS20; CYHR1; FAM219A; IDNK; C5; RP11-291L22.6; ZNF239; HNRNPH3; MYOF; PDLIM1; CWF19L1; SEC31B; OSBPL5; RPL27A; C11orf74; SMCO3; RP11-446N19.1; ATP8A2; RPL21; SPRY2; PABPN1; MIS18BP1; CDKN3; CASC4; COPS2; MIR3677; MAPK7; PGAP3; SNF8; RP11-159D12.2; KPNA2; RECQL5; RPL17; SERPINB5; OAZ1; YJEFN3; GMIP; ZNF793-AS1; CLASRP; GPR4; C20orf194; ZNF341; EIF2S2; DONSON; DLG3; MAGT1; RENBP |
| Cluster 10 | PRPF3; PPP2R5A; ANGEL2; TOMM20; SUPT7L; YIPF4; SOCS5; CCT4; PTCD3; C2orf47; NDUFAF3; RBM5; FAM208A; ZBTB11; GTPBP8; NCBP2; YTHDC1; TMA16; CCT5; CNOT6; MCUR1; NHLRC1; EIF2AK1; NDUFA4; ZSCAN21; MEST; PAXIP1; TNFRSF10A; UBE2V2; LYPLA1; ZNF517; CDC26; ODF2; SURF2; CCDC183-AS1; NRBF2; TBC1D12; TDRD1; CCDC90B; APPL2; UTP14C; EIF2AK4; RPS2; B3GNTL1; MED25; ZNF615; COMMD7; SNORA60; DPM1 |
| Cluster 11 | SAMD11; ATAD3A; FLAD1; SETD2; SLC25A36; AP2M1; ECE2; CTD-2194D22.3; ZNF622; RXRB; WBSCR27; RFC2; ZCWPW1; JHDM1D-AS1; ZNF746; EXOSC1; TCF7L2; MRPL17; TIMM10; SDHAF2; DDIT3; POLR1D; KDELC1; NFKBIA; KIF1C; UBALD2; RP11-672L10.6; MBD3; RPL10P15; UBL5; C19orf52; RPS16; VRK3; TFPT; ZNF837; CHMP4B; ARFGAP1; CSTB; TUBGCP6 |
| Cluster 12 | NOC2L; ENSA; E2F6; HMGB1P5; LYAR; RIPK1; MIR7111; RTN4IP1; FAM229B; PSMG3-AS1; IQCE; FBXL6; PIDD1; CFL1; LRFN4; TIMM8B; TRAPPC4; RPS2P5; HERC2P2; HAGH; ZNF597; RP1-59D14.5; ELP5; SUMO2; BIRC5; SYMPK; FTL; RP5-908M14.9; UCKL1; MIR647; TRMT2A; POLDIP3; NUP50-AS1; TRAPPC2; KIF4A; SLC6A8 |
| Cluster 13 | CD53; SLAMF8; CD48; FCGR2A; FCGR3A; LCP2; THBS2; SGPL1; TNNI2; LSP1; LMO2; APLNR; MPEG1; CD69; ASB2; TPSB2; ITGAM; CCL18; CCL3; CCL4L1; COL1A1; PLVAP; AMMECR1 |
